# Xenotropic MLV envelope proteins induce tumor cells to secrete factors that promote the formation of immature blood vessels

**DOI:** 10.1186/1742-4690-10-34

**Published:** 2013-03-27

**Authors:** Meera Murgai, James Thomas, Olga Cherepanova, Krista Delviks-Frankenberry, Paul Deeble, Vinay K Pathak, David Rekosh, Gary Owens

**Affiliations:** 1 Robert M. Berne Cardiovascular Research Center, University of Virginia, School of Medicine Charlottesville, Charlottesville, VA 22908, USA; 2 Department of Medicine, University of California, San Francisco, CA, 94143, USA; 3 Department of Biology, Mary Baldwin College, Staunton, VA, 24401, USA; 4 Viral Mutation Section, HIV Drug Resistance Program, Frederick National Laboratory for Cancer Research, National Cancer Institute, Frederick, MD, 21702, USA; 5 Myles H. Thaler Center for AIDS and Human Retrovirus Research and The Department of Microbiology, Immunology, and Cancer Biology, University of Virginia, Charlottesville, VA, 22908, USA

## Abstract

**Background:**

Xenotropic Murine leukemia virus-Related Virus (XMRV) is a γ-retrovirus initially reported to be present within familial human prostate tumors and the blood of patients with chronic fatigue syndrome. Subsequent studies however were unable to replicate these findings, and there is now compelling evidence that the virus evolved through rare retroviral recombination events in human tumor cell lines established through murine xenograft experiments. There is also no direct evidence that XMRV infection has any functional effects that contribute to tumor pathogenesis.

**Results:**

Herein we describe an additional xenotropic MLV, “B4rv”, found in a cell line derived from xenograft experiments with the human prostate cancer LNCaP cell line. When injected subcutaneously in nude mice, LNCaP cells infected with XMRV or B4rv formed larger tumors that were highly hemorrhagic and displayed poor pericyte/smooth muscle cell (SMC) investment, markers of increased metastatic potential. Conditioned media derived from XMRV- or B4rv-infected LNCaPs, but not an amphotropic MLV control virus infected LNCaPs, profoundly decreased expression of marker genes in cultured SMC, consistent with inhibition of SMC differentiation/maturation. Similar effects were seen with a chimeric virus of the amphotropic MLV control virus containing the XMRV *env* gene, but not with an XMRV chimeric virus containing the amphotropic MLV *env* gene. UV-inactivated XMRV and pseudovirions that were pseudotyped with XMRV envelope protein also produce conditioned media that down-regulated SMC marker gene expression *in vitro*.

**Conclusions:**

Together these results indicate that xenotropic MLV envelope proteins are sufficient to induce the production of factors by tumor cells that suppress vascular SMC differentiation, providing evidence for a novel mechanism by which xenotropic MLVs might alter tumor pathogenesis by disrupting tumor vascular maturation. Although it is highly unlikely that either XMRV or B4Rv themselves infect humans and are pathogenic, the results suggest that xenograft approaches commonly used in the study of human cancer promote the evolution of novel retroviruses with pathogenic properties.

## Background

Xenotropic Murine leukemia virus-Related Virus (XMRV) is a γ-retrovirus that was initially reported as present in human prostate tumors from patients with RNaseL inactivating polymorphisms [1-3] and in the blood of patients with chronic fatigue syndrome [2,4]. However, recent studies were unable to replicate these results [5-9]. A recent study by one of our laboratories [10], demonstrated that XMRV likely arose through recombination of two murine endogenous proviruses, named PreXMRV-1 and PreXMRV-2, as a consequence of xenograft experiments involving implantation of human tumor cells into nude mice followed by selection based on desired tumorigenic phenotypes including androgen independence, rapid anchorage-independent growth, and other properties. It now appears very likely that detection of XMRV in human tissues represented laboratory contamination and/or faulty PCR methodology [11-13].

The evidence that XMRV was generated as a consequence of studies aimed at elucidating the pathology of human disease is disturbing in that it highlights long feared dangers of use of xenograft tissues in clinical settings, including porcine valves [14,15]. Of even greater concern, the results support the idea that attempts to develop better therapeutic interventions might inadvertently promote the development of pathogenic viruses. However, the following observations refute this possibility: First, although xenotropic and polytropic MLVs have been described as far back as 1970 [16,17], as of yet there has been no validated evidence of human infection by this class of viruses. Second, despite intensive investigation of XMRV by many laboratories [1,18,19] there is no evidence that XMRV is capable of inducing transformation of cells [1,20], although there is recent evidence showing that XMRV infection of LNCaP cells resulted in modest increases in proliferation, and invasion of cells into Matrigel *in vitro* (*Pandhare-Dash et al.*[4,21]). Consistent with these findings, *Stieler et al.*[5,22,23] showed that shRNA-induced suppression of XMRV particle production reduced migration and the secretion of cytokines including osteopontin, CXCL14, IL13, and TIMP2 in the human prostate tumor cell line 22Rv1 *in vitro*, and resulted in decreased angiogenesis, reduced tumor size, and increased necrosis when cells were implanted in nude mice *in vivo*. Whether these differences can be directly ascribed to XMRV warrants further investigation, as this study does not compare these results to an uninfected cancer cell line control and nor identify how XMRV might induce functional changes.

Nevertheless, these results are of considerable interest since they suggest that infection of tumor cells with XMRV induces multiple changes that could possibly impact tumor pathogenicity, although presumably only in an experimental setting since there is no evidence it is capable of infecting human cells other than *in vitro* or in a xenograft. Given the remarkable plasticity of retroviruses, and the widespread use of xenograft approaches in the study of cancer, there are a number of critical unresolved questions: 1) While the combination of events that led to derivation of XMRV are extraordinarily rare [10,24,25], do similar XMRV-like viruses exist in other xenograft-derived cell lines due to selection processes common in these sorts of experiments? 2) Does XMRV infection of tumor cells impact tumorigenicity *in vivo*? 3) What are the mechanisms by which XMRV or XMRV gene products alter the functional properties of tumor cells in ways that may impact tumor pathogenesis? The studies described herein address these questions, and show that at least one other XMRV-like virus exists, and that the virus evolved the ability to infect human cells and to express gene products that impact tumor pathogenesis.

## Results

### Identification of an independent XMRV-like retrovirus derived from a mouse-human xenograft-derived prostate tumor cell line

**Figure 1:**
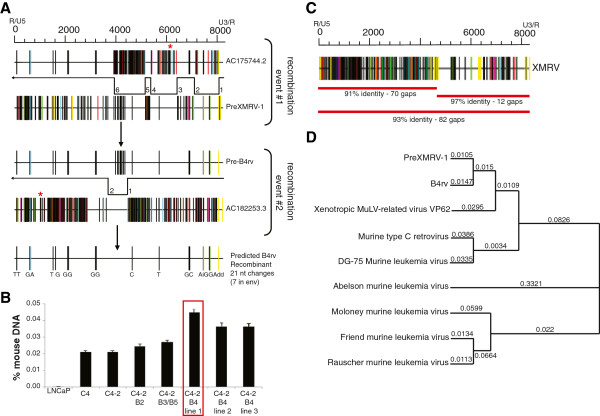
**Sequence and phylogenetic analysis of a novel xenotropic XMRV-like MLV B4rv, derived from the highly metastatic human prostate tumor xenograft-derived cell line C2-4 B4 **[16]**. A**. Hypermut plots for PreXMRV-1, Mus musculus chromosome Y BAC clones RP24-163J18 (AC175744.2) and RP24-320A8 (AC182253.3), Pre-B4rv and B4rv, where vertical lines indicate nucleotide mismatches relative to XMRV, demonstrating the likely proviral sequences from which B4rv originated. Red stars indicate a stop codon in *env* for AC175744.2 and a stop codon in *gag *for AC182253.3. Six template switches occurred between AC175744.2 and PreXMRV-1 to generate Pre-B4rv. Two template switches between Pre-B4rv and AC182253.3 generated the predicted B4rv recombinant. The predicted B4rv recombinant differs in sequence from B4rv by 21 nucleotide changes (18 nucleotide substitutions, one insertion (i) and two nucleotide deletions (d)). B4rv was identified based on representative difference analysis differential cloning comparing LNCaP human prostate tumor cells versus the C2-4B4 prostate tumor cell line indicated in (**B**). A schematic depicting the various LNCaP- and Xenograft-derived human prostate tumor cell lines is depicted in (Additional file 1: Figure S1). **B**. PCR for mouse-specific intracisternal A-type particle (lAP) long-terminal repeat sequences quantifying the relative amount of mouse DNA in the C4-2 B4 cell lines obtained from isolated genomic DNA of the sources indicated. The C4-2 B4 line outlined in red indicates the line from which B4rv was identified. **C**. Sequence alignment of B4rv and XMRV VP62 followed by a hypermut plot displaying the nucleotide mismatches of XMRV relative to B4rv. Red lines under the hypermut plot indicate regions of varying sequence identity between XMRV and B4rv, followed by a red line spanning the entire genome to indicate total sequence identity. **D**. Phylogenetic tree analysis of viruses in the MLV family, including xenotrophic MLVs, XMRV VP62 and B4rv, and proviral sequence PreXMRV-1.

To determine if additional XMRV-like viruses may have evolved independently as a result of mouse-human xenograft experiments, we performed differential screening of the LNCaP cell line versus C4-2 B4 cell line, a highly metastatic androgen-independent bone metastasis derived by *Thalmann et al.*[11,26] (Additional file 1: Figure S1a), using a high-stringency representative difference analyses method, more recently used routinely for differential cloning, but originally developed for high-sensitivity detection of low copy number retroviruses. Of major interest, we identified a putative xenotropic MLV retrovirus in the C4-2 B4 cell line that was absent from the original LNCaP cell line as well as the C4 and C4-2 cell lines that were also generated by *Thalmann et al.* Interestingly, whereas not all C4-2 B4 cell lines contained B4rv, all contained varying percentages of mouse DNA as measured by PCR for mouse-specific intracisternal A-type particle (lAP) long-terminal repeat sequences (Figure 1A), suggesting that B4rv arose from a rare set of events that occurred within the xenograft tumor rather than a common event that occurs in the co-culture of murine and human cells. B4rv was fully sequenced (Additional file 1: Figure S1b) and analyzed to identify the endogenous murine proviral sequences that contributed to its generation. These analyses indicated that the B4rv genome was derived from 2 independent recombination events between PreXMRV-1 and two different endogenous MLV proviral sequences on the mouse Y chromosome, representing 6 and 2 crossover junctions, respectively (Figure 1A). B4rv shares 93% overall sequence identity to XMRV, with only approximately 91% identity in the 5^′^ half of the genome and approximately 97% identity in the 3^′^ half (Figure 1C). Phylogenetic tree analyses demonstrated that while B4rv and XMRV share greater similarity to one another than other MLVs, B4rv represents a distinct sequence from that of XMRV (Figure 1D). Together, these data show that B4rv is a xenotropic MLV found in a human cell line derived through xenograft experiments in nude mice that is distinct in sequence and proviral origin from XMRV.

### XMRV- or B4rv-infected subcutaneous tumors were larger and exhibited defective vascular maturation

**Figure 2:**
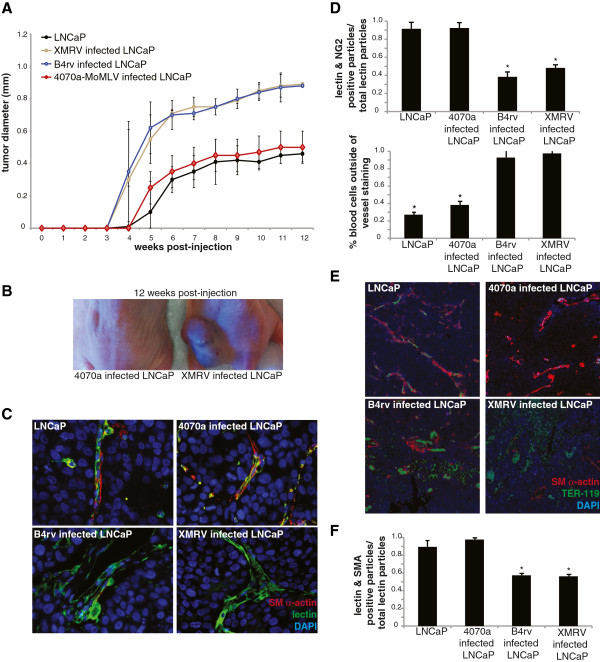
**Subcutaneous infection of XMRV- or B4rv-infected LNCaP cells results in increased tumor diameters, increased angiogenesis, decreased vascular SMC and pericyte investment, and increased hemorrhage *in vivo *. A**. Tumor diameter post-injection of LNCaP cells that were uninfected (control), or infected with either MoMLV-4070a, XMRV or B4rv demonstrating that tumors derived from XMRV- or B4rv-infected LNCaPs exhibited larger diameters starting at 6 weeks post-injection. N = 10/group **B**. Image of representative mice bearing tumors infected with MoMLV-4070a or XMRV at 12 weeks post-injection. **C**. Representative images of vessels in the tumors derived from uninfected LNCaPs or 4070a-MoMLV-, XMRV-, and B4rv-infected LNCaP cells at higher magnification, displaying deficient SMC-pericyte coverage in tumors derived from cells that were infected with either XMRV or B4rv. **D**. The percentage of pericytes (NG2-positive cells) associated with endothelial cells (isolectin positive cells), and SMCs (SM alpha-actin positive cells) associated with endothelial cells (isolectin positive cells), showing that tumors derived from XMRV- or B4rv-infected LNCaPs had significantly less pericyte and SMC association with blood vessels. Cell association was determined from lower magnification z-stacks of sections stained with isolectin, anti-NG2, anti-SM alpha-actin and DAPI (see Additional file 3: Figure S3) and quantified using the JaCoP plug-in [31] in Image J [34]. **E**. Representative images of tumors stained for red blood cells (RBCs) using antibodies against TER-119 in tumors derived from uninfected LNCaPs or 4070a-MoMLV-, XMRV-, and B4rv-infected LNCaP cells, showing increased hemorrhage in tumors derived from XMRV- or B4rv-infected LNCaPs. **F**. The percent of RBCs (TER-119 positive particles) found outside of blood vessels (isolectin staining).

To determine if infection of tumor cells by XMRV or B4rv confers any functional effects, we infected LNCaP cells with either XMRV or B4rv at similar infection levels (Additional file 1: Figures S1c, d) and implanted cells subcutaneously within Matrigel plugs in the flanks of nude mice and assayed tumor growth over 12 weeks. Our results showed that XMRV- or B4rv-infected LNCaP tumors grew to larger tumor diameters than uninfected LNCaP tumor cells (Figure 2A) or LNCaP cells infected with a control chimeric Moloney murine leukemia virus (MoMLV) containing the envelope gene of the amphotropic MLV 4070a to enable it to infect human cells (4070a) [14,27].

To determine mechanisms by which infection with XMRV and B4rv might increase tumor growth, we first tested if there were effects on proliferation or apoptosis. Staining and quantification of Ki-67 positive nuclei demonstrated no significant change in proliferation between tumors that resulted from XMRV- or B4rv-infected LNCaPs, and uninfected LNCaP tumors (Additional file 2: Figures S2a, c). These results were confirmed via *in vitro* cell counts (Additional file 2: Figure S2b). Staining of tumor sections for cleaved caspase-3 revealed a statistically significant increase in apoptotic cells within tumors derived from XMRV- or B4rv-infected LNCaP tumors as compared to uninfected or 4070a-infected LNCaP tumors (Additional file 3: Figure S3). As such, the observed increase in diameter of tumors with XMRV- or B4rv-infected LNCaP cells is not a direct function of a reduced apoptotic rate of tumor cells, although it is possible that the observed increase in apoptosis may contribute to tumor pathogenesis indirectly through generation of apoptotic products, and associated immune cell responses. Taken together, results indicate that the observed increases in size of XMRV- or B4rv-infected LNCaP tumors was not due to changes in the rate of tumor cell proliferation or apoptosis, thus implicating alternative mechanisms.

By gross examination, XMRV- or B4rv-infected LNCaP tumors appeared highly vascularized and hemorrhagic appearing nearly black at the surface of the tumor due to loss of blood into the interstitium (Figure 2B, Additional file 1: Figure S1f), suggesting that infection with these MLV viruses may have induced increased angiogenesis and defects in blood vessel maturation. We confirmed these findings by confocal analysis where staining with isolectin for endothelial cells, SM alpha-actin (Acta2) for vascular SMCs (Figure 2C) and NG2 for pericytes (Additional file 4: Figure S4, Additional file 5: Figure S5, Additional file 6: Figure S6, Additional file 7: Figure S7) showed an increase in vessel density (Additional file 1: Figure S1e) and a decrease in SMC and pericyte association with endothelial cells (Figure 2D, Additional file 1: Figure S1e). Staining for TER-119 for red blood cells and SM alpha-actin for SMCs (Figure 2E) showed a marked increase in hemorrhage, calculated as the percent of positive staining found outside of blood vessels (Figure 2F). Taken together, these results suggest that the increased size of XMRV- or B4rv-infected LNCaP tumors may be due at least in part to vascular leakage and deposition of blood products within the tumor stroma.

### B4rv- or XMRV-infected LNCaP cells secrete factors that promote HUVEC tube formation but inhibit differentiation of vascular SMCs *in vitro*

**Figure 3:**
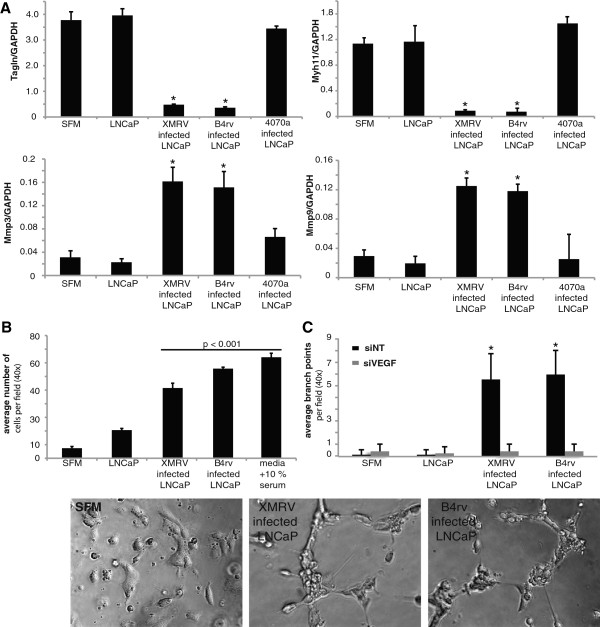
**Conditioned media from B4rv- or XMRV-infected LNCaP cells induce an immature SMC phenotype and promotes HUVEC tube formation *in vitro*. A**. QQ-rtPCR for expression of SMC marker genes *Tagln, Myh11*, and matrix metalloproteinase genes *Mmp3 *and *Mmp9*, in rat aortic SMCs after culture in tumour conditioned media (TCM) of uninfected LNCaPs, or 4070a-MoMLV-, XMRV-, and B4rv-infected LNCaP cells, demonstrating that SMCs down-regulate the expression of SMC marker genes when cultured in the TCM of LNCaPs infected with XMRV or B4rv but not those infected with MoMLV-4070a. TCM was generated by first applying viral particles of equivalent titers onto uninfected LNCaP cells for 24 hours, then culturing LNCaPs in serum-free media for 3 days. Media was collected and filtered through a 0.45μm pore to remove cellular debris, and then applied onto SMCs (see Methods). **B**. Migration of rat aortic SMCs across a transwell membrane in the presence of the TCM from uninfected LNCaPs, or 4070a-MoMLV-, XMRV-, and B4rv- infected LNCaP cells, showing that SMCs cultured in the TCM of LNCaPs infected by XMRV or B4rv exhibit increased migration as compared to SMC treated with TCM from uninfected LNCaPs. SMC culture media without serum (SFM) was used as a negative control, and culture media with 10% serum was used as a positive control. **C**. Tube formation assay of HUVECs cultured in Matrigel in the presence of the TCM from uninfected LNCaPs, and XMRV- or B4rv- infected LNCaP cells, demonstrating that HUVECs cultured in the presence of TCM from XMRV- or B4rv-infected LNCaPs form more tubes, based on number of branch points per field, than those cultured in the TCM of uninfected LNCaPs. HUVECs were treated with either siVEGF or a non-target siRNA control before plating in TCM and Matrigel, demonstrating that XMRV-infected LNCaP TCM induced HUVEC tube formation is VEGF-dependent.

Since XMRV- or B4rv-infected LNCaP tumors showed marked hemorrhage and defective perivascular cell coverage, we tested the hypothesis that viral infection induces tumor cells to produce soluble factors that contribute to defective differentiation of perivascular cells. We generated tumor conditioned media (TCM) from LNCaPs that were infected with XMRV, B4rv, or the amphotropic MLV MoMLV-4070a, and applied this TCM onto rat aortic SMCs *in vitro*. Infection of the rat aortic SMCs by virions shed into the TCM from infected LNCaP cells was not detected by PCR for integrated XMRV *gag* sequence in cellular genomic DNA isolated from SMCs 3 days following exposure to conditioned media (Additional file 8: Figure S8b). We examined the differentiation marker gene expression of these SMCs by q-rtPCR and found that TCM derived from LNCaP cells infected with either XMRV or B4rv, but not MoMLV-4070a or uninfected LNCaPs, induced profound suppression of expression of the SMC marker genes SM 22α (*Tagln*) and MHC (*Myh11*), but activated expression of *Mmp3* and *Mmp9* (Figure 3A). In contrast, direct application of XMRV or B4rv viral particles onto rat aortic SMCs did not result in suppression of SMC marker genes (Additional file 8: Figure S8a). TCM from XMRV- or B4rv-infected LNCaPs, but not from uninfected LNCaPs, also induced an increase in the migration of SMC across a transwell membrane (Figure 3B) that was ß1-integrin dependent (Additional file 8: Figure S8c). We also measured endothelial tube formation by plating human umbilical endothelial cells (HUVECs) in Matrigel mixed with the TCM from XMRV- or B4rv-infected LNCaP cells and found that there was an increase in VEGF-dependent tube formation as compared to HUVECs plated in Matrigel mixed with the TCM of uninfected LNCaP cells (Figure 3C, Additional file 8: Figure S8d). Taken together the above findings indicate that LNCaPs infected with XMRV or B4rv secrete factors that induce increased HUVEC tube formation, but a less differentiated/immature SMC phenotype. These results are consistent with results showing that infection with these two viruses also disrupted vascular maturation within subcutaneous tumors in our *in vivo* studies (Figures 2B-E, Additional file 4: Figure S4, Additional file 5: Figure S5, Additional file 6: Figure S6, Additional file 7: Figure S7).

### XMRV envelope proteins mediate inhibition of differentiation in vascular SMCs

**Figure 4:**
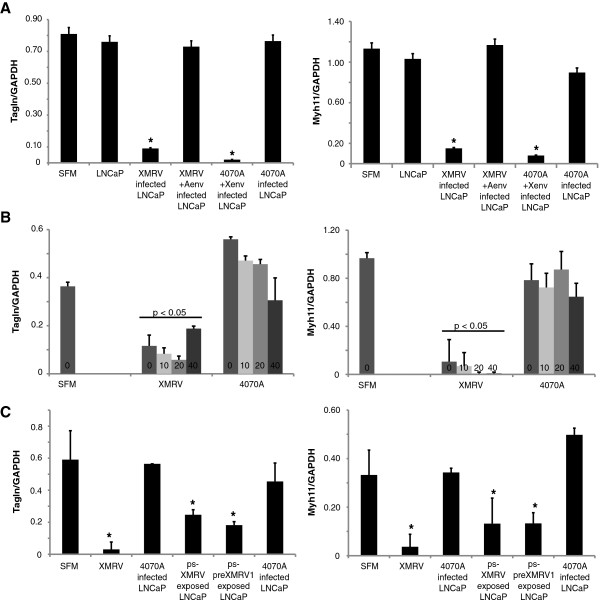
**Down-regulation of SMC marker genes in response to XMRV-infected LNCaP conditioned media is dependent on the presence of XMRV envelope proteins. A**. The *env* genes of 4070a and XMRV were swapped to form chimera retroviruses that were used to infect LNCaP cells in equal viral titers. XMRV-4070a-env was cloned by replacing the *env *gene of XMRV VP62 with the *env *gene of MoMLV-4070a. 4070a-XMRV-env was cloned by replacing the *env *gene of MoMLV-4070a with the *env *gene of XMRV VP62. Both chimera viruses (XMRV-4070a-env and 4070a-XMRV-env) represent fully infectious, replication competent viruses (Additional file 4: Figure S4). TCM was generated from the resulting tumor cells including uninfected LNCaPs, and MoMLV-4070a-, XMRV-, XMRV-4070a-env-, or 4070a-XMRV-env-infected LNCaP cells. Rat aortic SMCs were cultured in TCM for 72 hours followed by q-rtPCR analysis of expression of SMC differentiation marker genes including *Tagln *and *Myh11. ***B**. Q-rtPCR for expression of smooth muscle marker genes *Tagln* and *Myh11 *in rat aortic SMCs after culture in TCM of uninfected LNCaPs, LNCaps infected with replication competent viruses MoMLV-4070a or XMRV, or MoMLV pseudotyped with XMRV envelope, amphotropic envelope or PreXMRV-1 envelope and containing only the GFP gene, demonstrating that SMC marker gene down-regulation when cultured in the TCM of LNCaPs infected with XMRV is dependent on the presence of the XMRV env proteins*.***C**. Q-rtPCR for expression of SMC marker genes *Tagln *and *Myh11* in rat aortic SMCs after culture in the TCM of LNCaPs that were infected with XMRV or MoMLV-4070a viral particles that were exposed to 0, 10, 20 or 40 minutes of UV irradiation, demonstrating that LNCaP cells that are cultured with XMRV particles that are exposed to UV irradiation at doses that inactivate viral infectivity (Additional file 5: Figure S5) produce TCM that suppresses marker gene expression in rat aortic SMCs.

Because the TCM of MoMLV-4070a infected LNCaPs did not suppress SMC marker gene expression while that of XMRV- or B4rv-infected LNCaPs did (Figure 3A), we sought to investigate what gene products of the xenotropic MLVs contributed to suppression of SMC marker gene expression. Since xenotropic MLVs enter host cells via a receptor that is distinct from that of amphotropic MLVs, and since the *env* genes are homologous between XMRV and B4rv, we began by asking whether the xenotropic envelope gene products, which are responsible for mediating viral entry into host cells, were sufficient to induce the production of factors by LNCaP cells that suppress SMC marker gene expression. We substituted a portion of the XMRV genome that contains *env* into MoMLV-4070a to form a replication competent chimera virus (Additional file 9: Figures S9a, c) termed X-Aenv and found that the presence of *env* from XMRV was sufficient to induce the production of TCM by infected LNCaP cells that suppressed SMC marker gene expression to a degree equivalent to XMRV or B4rv (Figure 4A). In contrast, TCM from LNCaP cells infected with a chimeric virus consisting of XMRV containing *env* of MoMLV-4070a (Additional file 9: Figures S9b, c) did not exhibit SMC differentiation suppressing activity (Figure 4A).

To determine if viral gene expression is necessary to confer this phenotype, we exposed XMRV or MoMLV-4070a viral particles to UV irradiation prior to applying them on LNCaP cells to generate TCM. Although only 10 minutes of exposure to UV irradiation was needed to ablate the infectivity of either virus (Additional file 9: Figure S9e), the TCM of LNCaPs exposed to XMRV viral particles that were UV irradiated for 10 minutes suppressed SMC marker gene expression, with no significant difference when compared to the TCM of LNCaPs infected with XMRV not exposed to UV irradiation (Figure 4B). TCM from LNCaPs exposed to MoMLV-4070a viral particles that were irradiated at any dose was unable to suppress SMC marker gene expression, just as was observed using TCM from LNCaPs infected with MoMLV-4070a that was not irradiated.

To further explore whether the presence of xenotropic MLV envelope proteins at the cell surface was sufficient to confer this phenotype, we generated XMRV pseudotyped MoMLV viral particles that were only capable of expressing GFP, and not any retroviral genes (Additional file 9: Figure S9d). In addition, PreXMRV-1-pseudotyped MoMLV viral particles were also produced and included in this experiment due to the sequence similarity of this proviral sequence to B4rv in the *env* gene (Figure 1A). TCM from LNCaP cells exposed to XMRV or PreXMRV1 pseudotyped viral particles, but not from LNCaP cells exposed to viral particles pseudotyped with an amphotropic MLV envelope, was able to suppress SMC marker gene expression (Figure 4C). Taken together the results of the preceding experiments provide compelling evidence that the *env* gene products of XMRV and B4rv are sufficient to induce the production of factors by infected tumor cells into conditioned media that modulate SMC marker gene expression. These data suggest that xenotropic MLV envelope proteins assembled on viral particles, independent of other xenotropic MLV genes, induce the production of soluble factors by tumor cells that suppress SMC marker gene expression, resulting in defective blood vessel maturation and increased vessel density.

## Discussion

Our results reveal the presence of a novel xenotropic MLV that, like XMRV, was likely generated through the recombination of endogenous murine proviral sequences via xenograft experiments in nude mice. We postulate that such recombination events may be extremely rare and yet the generation of similar viruses may be enhanced as a consequence of extraordinary selection pressures inherent in typical xenograft and culture experiments. We further show that defective blood vessel formation is a functional consequence of infection by these xenotropic MLVs, resulting in larger tumors that possess greater numbers of blood vessels that are immature and hemorrhagic. Remarkably, our *in vitro* results indicate that exposure to the xenotropic MLV envelope proteins is sufficient to induce tumor cells to produce factors that alter vascular SMCs to an immature phenotype, suggesting a novel mechanism by which xenotropic viral infection of tumor cells leads to the formation of immature blood vessels.

Disrupted tumor vascular maturation has long been equated with high rates of tumor cell shedding and metastasis [16,28]. Highly hemorrhagic tumors that contain an abundance of dilated, immature blood vessels poorly invested with vascular SMCs and pericytes exhibit increased metastatic potential [18,29]. Consistent with this possibility, *Ramaswamy et al.*[20,30] used microarray analysis of over 800 human solid tumors and metastases and demonstrated that 4 of the 9 genes whose down-regulation is highly predictive of tumor metastasis encode markers of differentiated SMC/pericytes, suggesting that a lack of differentiated SMC/pericytes within tumor biopsy samples is highly predictive of tumor metastatic potential. Immature vascular networks are also highly inefficient for blood delivery and exhibit impaired delivery of chemotherapeutic agents, resulting in higher levels of agents in normal versus tumor tissues [21], which is also thought to contribute to the poor overall efficacy of anti-angiogenic therapies due to counteracting effects of combined therapies. The mechanisms that contribute to incomplete maturation of tumor vessels are poorly understood, although it may result in part from a hyper-VEGF state, as supported by studies in both animal models and human clinical trials showing at least partial normalization of vessel structure with anti-VEGF therapies [22,23].

Although neither XMRV nor B4rv are likely to infect humans and contribute to tumor pathogenesis, our observations that xenotropic MLV envelope proteins induce signalling that results in an immature vascular phenotype provide a novel mechanism by which viral gene products might promote tumor pathogenesis. However, there are a number of key unresolved questions: First, what are the mechanisms by which the XMRV envelope proteins interact with tumor cells? Although the known viral entry receptor XPR1 has been shown to mediate entry of XMRV into some cells [24,25], there is also recent evidence that XMRV can infect cells that do not express XPR1 [26]. Indeed, we have been unable to inhibit the effects of XMRV envelope protein in suppressing SMC differentiation by siRNA-induced inhibition of XPR1 (Additional file 8: Figure S8e). As such, the effects of XMRV envelope protein observed in the present studies appear to be mediated by a receptor or membrane protein or moiety other than XPR1. Based on our data showing an increase in VEGF-dependent endothelial tube formation in the presence of conditional media from B4rv- or XMRV-infected tumor cells (Figure 3C, Additional file 8: Figure S8d), this receptor might be one of a wide range of cell surface molecules that, when activated, may result in the release of VEGFs. Of interest, our observation that production of soluble factors that repress vascular SMC differentiation persisted in LNCaP cells chronically infected with B4rv or XMRV suggests that the envelope protein receptor may not be down-regulated or de-sensitized despite chronic exposure to ligand, and/or that there are positive feedback mechanisms that result in continual regeneration of receptor. Second, what are the rate-limiting mechanisms that prevent xenotropic and/or polytrophic MLVs from infecting humans? Of significance, productive infection by XMRV or B4rv of human cells *in vitro* appears to be limited to cells that contain defective defences to retroviruses. Indeed, the prostate cancer LNCaP cell line used herein lacks XMRV-restricting APOBECs [27] and contains a deletion mutation of one allele of RNaseL [28], a gene critical in the innate immune response to retroviruses. Inactivating polymorphisms of RNaseL are highly associated with familial prostate cancer in young males, and the hypothesis that such deficiencies in innate immunity leaves one vulnerable to a pathogenic retrovirus was the basis for the study that lead to the discovery of XMRV. Since then, any association of XMRV and human infection has been invalidated. However, a critical question is whether utilization of RNaseL defective tumor cells to generate xenograft derived cell lines might allow for the evolution of retroviruses that ordinarily cannot escape cellular immune defences. Third, how does binding of envelope protein to XPR1 or alternative receptor on tumor cells induce production, and/or release of soluble factors that disrupt tumor vascular maturation?

Signalling through retroviral envelope proteins has been shown in other disease models, notably by the Jaagsiekte sheep retrovirus (JSRV) whose envelope proteins cause ovine pulmonary adenocarcinoma through the activation of multiple signaling pathways including the phosphoinositide-3-OH kinase (PI3K)/Akt, mitogen-activated protein kinase (MAPK) signaling cascades, binding/degradation of hyaluronidase 2 (Hyal2), and activation of the RON receptor [29]. Degradation products of Hyal2 in particular have been associated with an increase in angiogenesis in the context of xenograft prostate cancer models, outside of the context of JSRV infection. A recent study reports an increase in pro-angiogenic factors within tumors infected with JSRV, particularly VEGF-C and PDGFR-alpha. However the role of the envelope protein in factor production has yet to be elucidated. Other examples of retroviral envelope induced cellular signaling include the Friend spleen focus-forming virus (SFFV), mouse mammary tumor virus (MMTV), enzootic nasal tumor virus (ENTV), and avian hemangioma retrovirus (AHV), all in which envelope proteins contribute to, or induce, transformation [30]. Although previous work by other labs demonstrates that XMRV does not induce transformation [1-3], no work to date has shown whether xenotropic MLVs as a class may activate signaling pathways that promote a pro-tumorigenic environment through indirect mechanisms, including through inducing the secretion of pro-angiogenic factors. This is especially important to examine given that unlike other MLVs that arise in a more organic fashion, all xenotropic MLVs identified to date have been found in the context of xenograft experiments that establish cell lines in the laboratory setting [2,31], providing a unique and specific selection on this class of MLV. Indeed, given that XMRV and B4rv evolved independently but elicit very similar functional consequences in tumor cells, it is interesting to speculate that there is a unique aspect to xenograft experiments that selects for retroviruses that induce larger, more hemorrhagic tumors.

## Conclusions

In summary, the studies described here have identified a second independent xenograft-derived MLV retrovirus designated B4rv that, like XMRV, has acquired the capability of infecting human tumor cells *in vitro*. Moreover, we provide novel evidence that infection of tumor cells with either XMRV or B4rv results in tumors that are larger and which exhibit multiple changes consistent with disruption of tumor vascular maturation including decreased perivascular cell coverage, and increased hemorrhage. Although it is extremely unlikely that XMRV or B4rv have, or could infect humans [6-9,32], results herein raise the possibility that additional XMRV-like viruses may exist, or could evolve, that contain gene sequences that impact tumor pathogenesis, and of greatest concern might also acquire the ability to infect humans.

## Methods

### Mice

BALB/cAnNCr-nu /nu male mice were obtained at 6 weeks of age from the NCI Frederick Animal Production Program and were handled according to protocols approved by the UVA Institutional Animal Care and Usage Committee guidelines. Each mouse was subcutaneously injected in both flanks with 1x10^6 cells suspended in a 50/50 mixture of PBS and Matrigel. Tumor growth was measured with calipers once per week post-injection, every 2 days once tumor growth is apparent and daily as tumors reach near maximum tumor burden. Mice were euthanized by CO_2_ asphyxiation when total tumor burden was reached, as defined as 10% of total body weight, then perfused via the left ventricle with 5 mL PBS followed by 10 mL 4% paraformaldehyde and an additional 5 mL PBS. Tumors and various organs were dissected and either fixed in 4% paraformaldehyde prior to embedding in paraffin, or stored in PBS for no more than 1 hour before embedding in low-melt agar.

### Immunofluorescent staining

Paraffin blocks were sectioned at 10 μm thick. Sections were de-paraffinized and rehydrated in xylene and ethanol series. After antigen retrieval (antigen retrieval solution, Vector), sections were blocked with Fish Skin Gelatin/PBS (6 g/L) containing 10% of goat or horse serum for 1 hour at room temperature. An endogenous mouse IgG blocking consisting in incubation of mouse tissue sections with unconjugated Fab Fragment Goat anti-mouse IgG (H+L) (Jackson ImmunoResarch Labs) for 1 hour at room temperature were performed. Slides were incubated with the following antibodies: isolectin g5-IB_4_ Alexa-647 conjugate (5 μg/mL, Invitrogen), mouse monoclonal SM α-actin-Cy5 (4.4 μg/mL, Sigma Aldrich), rabbit polyclonal NG2 (5 μg/mL, Millipore) and rat monoclonal TER-119 (5 μg/mL, Santa Cruz). The secondary antibody used to detect NG2 staining was donkey anti-rabbit conjugated to Alexa-488 (5 ug/mL, Abcam); to detect TER-119 staining donkey anti-rat conjugated to Alexa-488 (5 μg/mL) was used. For blood vessel density measurements, tissues that were embedded in low-melt agar were sectioned at 50 μm thick and permeablized in Eppendorf tubes with 0.02% saponin. Sections were stained with isolectin g5-IB_4_, SM alpha-actin-Cy5 and NG2 as described above, then transferred to slides, counter-stained with DAPI and coverslipped for imaging.

### Image acquisition and analysis

Images were acquired with Olympus BX41 fitted with a Q imaging Retiga 2000R camera. Image acquisition was performed with the Q Capture Pro software (Media Cybernetics & QImaging Inc). Settings were fixed at the beginning of both acquisition and analysis steps and were unchanged. Brightness and contrast were lightly adjusted after merging. Image analysis was performed with Image J [10,33] using the JACoP plugin [12,13,34] for quantification of percent cell coverage and manual analysis for vessel density. Confocal images were acquired using a Zeiss LSM700 scanning confocal microscope with 405nm, 488nm, 555nm, and 637nm solid-state lasers. Analysis of confocal images was completed using Zeiss Zen 2009 software.

### Cell culture

LNCaPs and C42-B4s were cultured in T-media (Gibco), supplemented with Fetal Bovine Serum (5%), and Penicillin-Streptomycin (100 U/mL, Gibco). Rat aortic SMCs were isolated and cultured as previously described [15,35] and were cultured in growth medium (DMEM/F12, Gibco) supplemented with Fetal Bovine Serum (10%), L-glutamine (1.6 mM, Gibco) and Penicillin-Streptomycin (100 U/mL, Gibco). HUVECs were cultured on dishes coated with 0.1% gelatin in M-199 media supplemented with Fetal Bovine Serum (10%).

### Viruses

The XMRV clone VP62 plasmid was obtained from Dr Robert Silverman [17,36]. The MoMLV-4070a plasmid was obtained from Dr Alan Rein [1,14,19]. B4rv was isolated and sequenced from the C42-B4 cell line obtained from Dr Sally Parsons (University of Virginia). All viruses were produced by transfection of 293T cells with appropriate plasmid, harvesting the culture medium 12 to 72 hours later, and filtering the medium through 0.45-μm-pore low-protein-binding filters to remove cells and debris. Viruses were stored at -80°C. Viral titers were established by infection of naïve LNCaPs plated on glass coverslips within 6-well plates with serial dilutions of viral stocks for 24 hours, followed by formalin fixation and staining for gag proteins using rabbit polyclonal gag antibody (5 μg/mL, Abcam) followed by the secondary donkey anti-rabbit conjugated to Alexa-488 (5 ug/mL, Abcam).

### Construction of amphotropic/xenotropic chimera viruses

The chimera viruses of VP62 containing the *env* of 4070a and MoMLV-4070a containing the env of VP62 were constructed using gene splicing by overlap extension (gene SOEing) (see Table 1 for primer sequences). Viruses were generated from these two resulting plasmids by transfection into 293T cells and harvest of the culture medium 12 to 72 hours later.

**Table 1:** Primer sequences

**Primer name**	**Sequence**	**Used to generate data for figure:**
IAP-F [8]	5^′^-ATAATCTGCGCATGAGCCAAGG-3^′^	1
IAP-R [8]	5^′^-AGGAAGAACACCACAGACCAGA-3^′^	1
Tagln F	5^′^-GCATAAGAGGGAGTTCACAGACA-3^′^	3, 4, S11
Tagln R	5^′^-GCCTTCCCTTTCTAACTGATGATC-3^′^	3, 4, S11
Myh11 F	5^′^-CAGTTGGACACTATGTCAGGGAAA-3^′^	3, 4, S11
Myh11 R	5^′^-ATGGAGACAAATGCTAATCAGCC-3^′^	3, 4, S11
1F [33]	5^′^-GCGCCAGTCATCCGATAGACT-3^′^	S2
K1R [33]	5^′^-AAGGCTTTATTGGGAACACG-3^′^	S2
MoA 5 bbone	5^′^-GCGCCAGTCCTCCGATTGAC-3^′^	S13
XMRV adapt MoA	5^′^ -TGAGAACGCTGGACTTTCCATC	S13
5bbone R	GATGTTAGGCCATTAAGGAG-3^′^	
XMRV adapt MoA	5^′^-AACTCCTCCGGCCGGAACAGC	S13
env F	ATGAGACAGCGGACCCGGACT-3^′^	
MoA adapt XMRV	5^′^-GAAACTGAATAAAATCTTTTAT	S13
env R	GGCTCGTACTCTATAGGCTT-3^′^	
MoA adapt XMRV	5^′^-GAAGAAGTGGAATCACGTGA	S13
3bbone F	ATAGATAAAATAAAAGATTTTA-3^′^	
MoA 3bbone R	5^′^-TGCAACTGCAAG	S13
	AGGGTTTATTGG-3^′^	
XMRV 5bbone F	5^′^-GCGCCAGTCCTCCGATTGAC-3^′^	S14
MoA adapt XMRV	5^′^-CTCCTTAATGGCCTAACATCG	S14
env F	ATGGAAAGTCCAGCGTTCTCA-3^′^	
MoA adapt XMRV	5^′^-GAAACTGAATAAAATCTTTTA	S14
env R	TGGCTCGTACTCTATAGGCTT-3^′^	
XMRV adapt MoA	5^′^-TAAAATCTTTTATTTTATCTA	S14
env R	TTCACGTGATTCCACTTCTTC-3^′^	
XMRV 3bbone F	5^′^-AAGCCTATAGAGTACGAGCCA	S14
	TAAAAGATTTTATTCAGTTTC-3^′^	
XMRV 3bbone R	5^′^-TTGCAAACAGCAAAAGGCTTT-3^′^	S14
XPR1 F [35]	5^′^- TAATTCAGAAGAATCAGGAATT-3^′^	S16
XPR1 R [35]	5^′^-CGAGTGACCTCGTTCTTTG-3^′^	S16

### Infection of LNCaP cells and TCM generation

LNCaP cells were seeded into 6-well plates at 2×10^4 cells per well, and the medium was replaced 1 day later. Two days after cell seeding, ~10^6 infectious units was added to each well with 10 μg/ml Polybrene to increase the infection efficiency. The medium was replaced 1 day after infection with serum free medium. Three days after infection, culture supernatant was harvested and passed through 0.45-μm-pore low-protein-binding filters to remove cells and debris and then used as TCM.

### SMC marker gene expression by q-rtPCR

Rat aortic SMCs were seeded into 6-well plates at 2×10^4^ cells per well, and allowed to adhere for 1 day. Culture media was then removed, cells were briefly washed with PBS and TCM generated from infected LNCaP cells as described above was placed in each well (2 mL per well). SMCs were cultured for another 3 days and then total RNA was isolated using the Trizol reagent (Invitrogen) according to the protocol of the manufacturer. One microgram of RNA was reverse transcribed with iScript cDNA synthesis kit (BioRad). Q-rtPCR was performed by iCycler technology (BioRad) for SMC marker genes *Tagln*, *Myh11*, *Acta2* as well as the genes *Mmp3* and *Mmp9*. Expression of genes was normalized to *Gapdh* levels. See Table 1 for primer sequences.

### SMC migration

Cell migration assays were performed on Millipore MultiScreen-MIC plates with 8 μm pores. Rat aortic SMCs were grown to 70% confluence and then switched to serum-free media. A cell suspension (1×10^5^ cells/mL, 150 μL) was added to the upper well in serum-free media containing 0.1% BSA (Sigma). TCMs from infected or uninfected LNCaPs were added to the bottom chambers. For all experiments 5 μg/ml fibronectin (Sigma) was added to the bottom chamber in serum-free media with 0.1% BSA. The chambers were incubated at 37°C in a CO_2_ incubator for 16 hours, and fixed in 4% formaldehyde. The cells that did not migrate (remained on top of the membrane) were removed from the upper wells and the invaded cells were stained with 0.2% Crystal Violet solution in 7% ethanol. Cells from 8–10 randomly chosen high-power fields (magnification x20) on the lower surface of the filter were counted.

### HUVEC tube formation and siVEGF transfection

50 μL of Matrigel was plated in each well of a 96-well plate on ice using pre-chilled pipette tips, avoiding the generation of any bubbles. The plate was then incubated for 30 minutes at 37°C to allow the Matrigel to solidify. HUVECs were suspended at a density of 1.5x10^5 cells/mL in the TCM of infected LNCaPs generated as described above. 150 μL of HUVECs in TCM was pipetted on top of the solidified Matrigel in the 96-well plate, and the plate was incubated for 8 hours at 37°C. Endothelial tube branch points from 8–10 randomly chosen high-power fields (magnification ×20) were counted.

### Statistics

Values are expressed as means ± s.e.m. q-rtPCR, migration and endothelial cell tube formation experiments were performed in triplicate. Comparison between groups was tested using non-parametric ANOVA test the Statistical Analysis System (SAS, version 9.2, Cary, NC). A value of P≤0.05 was considered significant.

## Competing interests

The authors declare no affiliations, funding or financial holdings that might be perceived as affecting the objectivity of this paper.

## Authors’ contributions

MM conceived the overall project, designed and executed studies, analysed data, constructed figures, and wrote the paper; JT discovered B4rv by performing screening of LNCaP cell lines by representative difference analyses; JT and PD generated the initial concept for the project and performed initial preliminary experiments; OC assisted in the design and implementation of migration and tube formation experiments; VP, KD-F, and DR analysed sequence data, assisted in experimental validation techniques and provided valuable experimental tools particularly with respect to virology. GKO provided overall supervision of the studies, aided M.M. in conceiving overall experimental strategies, and played a major role in writing the paper. All authors read and approved the final manuscript.

## Supplementary Material

Additional file 1: Figure S1 Schematic describing the generation of xenograft derived cell lines (*Thalmann et al.*[16]), validation of xenotropic MLV infection and vascular effects *in vivo*. (A) Adapted from Thalmann et al.[16] describing the generation of the C2-4 B4 cell line from which B4rv was discovered.LNCaP cells and a human bone fibroblast (MS) cell line, were injected into a nude mouse to generate a primary subcutaneous tumor, after which the mouse was castrated. The subsequent androgen independent tumor was established as the C4 cell line. An additional mouse was castrated and injected with C4 and MS cells to generate the C4-2 cell line. The C4-2 cell line was injected into additional mice to generate bone metastasis cell lines including C4-2 B2, C4-2 B3/B5 and the C4-2 B4. (B) The B4rv genome represented in red followed by open reading frames (ORFs) as black arrows.ORFs were determined and drawn using Gene Construction Kit(2), with a minimum ORF length of 250 base pairs, ‘ATG’ starting codon and searching only the top strand (5’ to 3’). The B4rv genome was isolated from genomic DNA of infected C4-2 B4 cell line via PCR using primers described in Urisman et. Al.(3) (see Table 1) and sequenced on an ABI 3730 DNA Analyzer, 750-1000 base pairs at a time. Overlapping sequences were assembled using the Geneious software(4) package. (C) In vitro staining with an antibody to MLV-gag, in 4070a-, B4rv- or XMRV-infected LNCaP cells infected with 1x106 infectious units, and no gag staining of the control LNCaP cells. (D) Reverse transcriptase activity in 4070a-, B4rv- or XMRV-infected, or uninfected LNCaP cells. (E) Quantification of tumor sections (n=10) stained with an endothelial cell specific lectin, alpha-SMA antibody, NG2 antibody. Measurements were taken by determining the percent of each field that was positive for signal using ImageJ(5). (F) A representative image of nude mice with tumors resulting from injection of LNCaP cells infected with 4070a (left) or XMRV (right) at 12 weeks post-injection. Click here for file

Additional file 2: Figure S2 LNCaP cells infected with MoMLV-4070a, B4rv or XMRV do not exhibit an increase in proliferation above uninfected LNCaP cells. (A) Representative Ki-67 staining of paraffin-embedded tumor sections resulting from subcutaneous injection of LNCaP cells infected with either 4070a, B4rv or XMRV into nude mice. (B) Quantification of in vitro proliferation assay where LNCaP cells infected with 4070a, B4rv or XMRV and the uninfected LNCaP control were plated at 1x10^6 ^cells per well, grown for 24 hours, then stained with crystal violet and counted at 20x. Results represent an average of 3 fields observed per well, 2 wells per group. (C) Quantification of *in vivo *Ki-67 staining displayed in (A), where positive nuclei were counted from sections of 10 tumors per group. Click here for file

Additional file 3: Figure S3 LNCaP cells infected with MoMLV-4070a, B4rv or XMRV exhibit an increase in apoptosis compared to uninfected LNCaP cells. (A) Representative cleaved caspase-3 staining of paraffin-embedded tumor sections resulting from subcutaneous injection of LNCaP cells infected with either 4070a, B4rv or XMRV into nude mice. (B) Quantification of *in vivo* cleaved caspase-3 staining displayed in (A), where positive nuclei were counted from sections of 10 tumors per group. Click here for file

Additional file 4: Figure S4 Representative confocal image of an uninfected LNCaP tumor stained for vascular cell markers. Merged confocal image showing staining of a paraffin-embedded tumor section resulting from subcutaneous injection of uninfected LNCaP cells into a nude mouse, shown at 20x. Insets are shown at 120% digital zoom using the Zeiss Zen 2009 software. SM α-actin, an SMC marker is shown in red, NG2, a pericyte marker, is shown in green, isolectin for endothelial cells is shown in magenta and nuclear stain DAPI is shown in blue. Click here for file

Additional file 5: Figure S5 Representative confocal image of a MoMLV-4070a infected LNCaP tumor stained for vascular cell markers. Merged confocal image showing staining of a paraffin-embedded tumor section resulting from subcutaneous injection of 4070a infected LNCaP cells into a nude mouse, shown at 20x. Insets are shown at 120% digital zoom using the Zeiss Zen 2009 software. SM α-actin, an SMC marker is shown in red, NG2, a pericyte marker, is shown in green, isolectin for endothelial cells is shown in magenta and nuclear stain DAPI is shown in blue. Click here for file

Additional file 6: Figure S6 Representative confocal image of a B4rv infected LNCaP tumor stained for vascular cell markers. Merged confocal image showing staining of a paraffin-embedded tumor section resulting from subcutaneous injection of B4rv infected LNCaP cells into a nude mouse, shown at 20x. Insets are shown at 120% digital zoom using the Zeiss Zen 2009 software. SM α-actin, an SMC marker is shown in red, NG2, a pericyte marker, is shown in green, isolectin for endothelial cells is shown in magenta and nuclear stain DAPI is shown in blue. Click here for file

Additional file 7: Figure S7 Representative confocal image of an XMRV infected LNCaP tumor stained for vascular cell markers. Merged confocal image showing staining of a paraffin-embedded tumor section resulting from subcutaneous injection of XMRV infected LNCaP cells into a nude mouse, shown at 20x. Insets are shown at 120% digital zoom using the Zeiss Zen 2009 software. SM α-actin, an SMC marker is shown in red, NG2, a pericyte marker, is shown in green, isolectin for endothelial cells is shown in magenta and nuclear stain DAPI is shown in blue. Click here for file

Additional file 8: Figure S8 B4rv or XMRV infection of tumor cells promotes the production of pro-angiogenic soluble factors in vitro, but does not infect vascular SMCs, nor directly suppresses SMC marker gene expressions (A) Q-rtPCR results of rat aortic SMCs *Tagln* and *Myh11* gene expression levels after exposure to XMRV viral particles at a range of concentrations (x-axis). (B) Gel electrophoresis image of PCR for an MLV-gag sequence, using genomic DNA from uninfected LNCaP cells, B4rv treated LNCaP cells, XMRV treated LNCaP cells and XMRV treated SMCs. PCR for GAPDH was used as a loading control. (C) Quantification of SMC migration assay where conditioned media was incubated with a blocking antibody to ß1 integrins or RGD peptides prior to plating under transwell membranes. Membranes were stained with crystal violet 16 hours post-incubation and counted at 40x. (D) Effects of TCM on tube formation by human umbilical cord endothelial cells (HUVEC) with and without addition of a VEGFR1/VEGFR2 inhibitor to the TCM prior to incorporation in matrigel plugs. Endothelial tube branch points from 8–10 randomly chosen high-power fields (magnification x40) were counted. (E) Q-rtPCR results of rat aortic SMCs *Tagln, Myh11* and *Xpr1* gene expression levels after exposure to conditioned media of LNCaP cells treated first with siRNA to XPR1 or a non-targeted control, then XMRV, 4070a, chimeric viruses 4070a-Xenv or XMRV-Aenv, or pseudovirions expressing GFP and coated with the envelope proteins of either XMRV or 4070a (psXMRV, ps 4070a). Click here for file

Additional file 9: Figure S9 Rat aortic SMCs are not infected by XMRV in vitro, and direct application of viral particles onto SMCs in culture does not result in the suppression of smooth muscle marker gene expression. (A) Schematic depicting 4070a-Xenv, blue representing the sequence from MoMLV-4070a, and red representing the sequence from XMRV VP62 and black arrows representingORFs. ORFs were determined and drawn using Gene Construction Kit(2), with a minimum ORF length of 250bp, ‘ATG’ start codon and searching only the top strand (5’ to 3’). The 4070a-Xenv chimera was constructed by replacing the env gene of MoMLV-4070a with the env gene of XMRV VP62 via overlap extension (see Table 1) and sequenced at the University of Virginia DNA Sciences Core on an ABI 3730 DNA Analyzer. Overlapping sequences were assembled using the Geneious software(4) package. (B) Schematic depicting XMRV-Aenv,blue representing the sequence derived from MoMLV-4070a, red representing the sequence derived from XMRV VP62 and black arrows representingORFs. ORFs were determined and drawn using Gene Construction Kit(2), with a minimum ORF length of 250bps=, ‘ATG’ start codon and searching only the top strand (5’ to 3’). The XMRV-Aenv chimera was constructed by replacing the env gene of XMRV VP62 with the env gene of MoMLV-4070a via overlap extension (see Table 1) and sequenced at the University of Virginia DNA Sciences Core on an ABI 3730 DNA Analyzer, 750-1000 base pairs at a time. Overlapping sequence contigs were assembled using the Geneious software package(4). (C) In vitro staining for MLV-gag, demonstrating gag protein production in 4070a-, XMRV-, 4070a-Xenv-, or XMRV-Aenv-infected LNCaPs, and no gag staining of control LNCaPs. (D) GFP signal overlayed on bright-field images of LNCaP cells in vitroexposed to MLV viral particles pseudotyped with the envelope of 4070a, pre-XMRV1 or XMRV for 24 hours. (E) Reverse transcriptase detection demonstrating absence of activity by 4070a or XMRV viral particles that were UV irradiated for 10, 20 or 40 minutes. Viral particles exposed to 0 minutes UV irradiation demonstrate reverse transcriptase activity. Click here for file
