# Supplementary figures and images for: Xenotropic MLV envelope proteins induce tumor cells to secrete factors that promote the formation of immature blood vessels
Source: Retrovirology. 2013 Mar 27;10:34. doi: 10.1186/1742-4690-10-34 (PMC3681559; doi:10.1186/1742-4690-10-34)

**Fig. S1**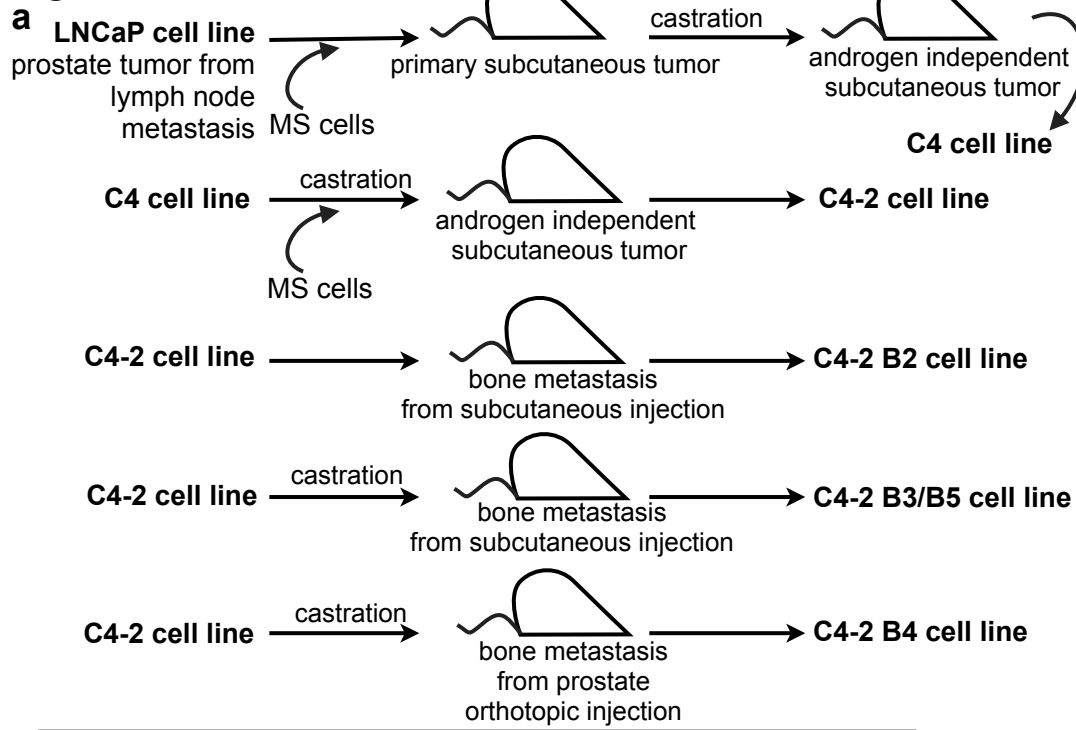

adapted from Thalmann et al. Prostate (2000) vol. 44 (2) pp. 91-103 Jul 1;44(2)

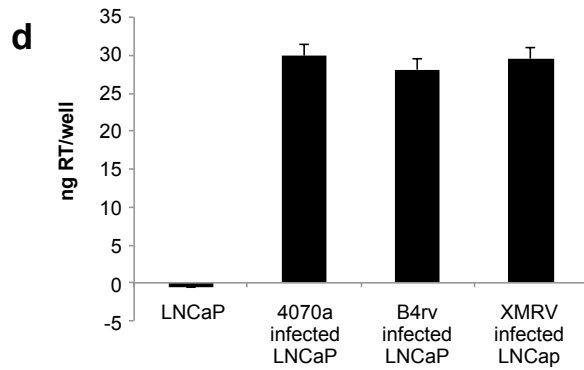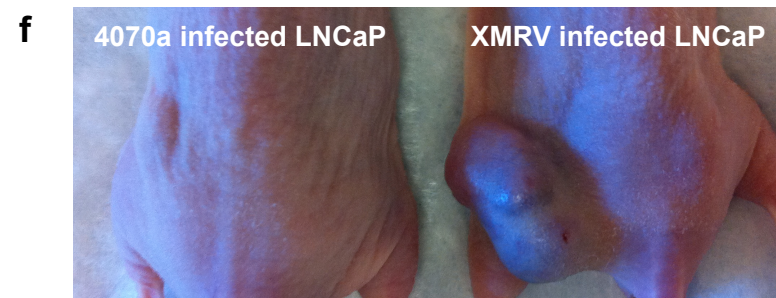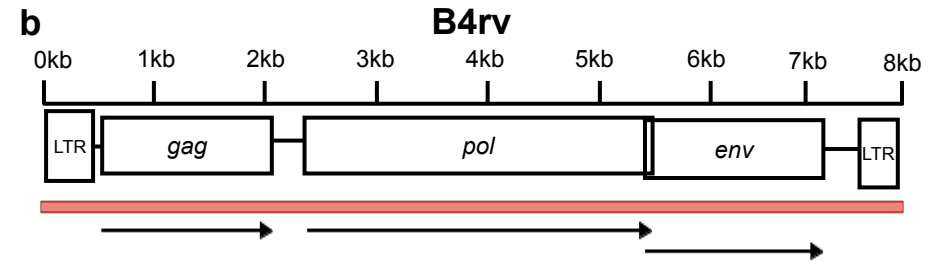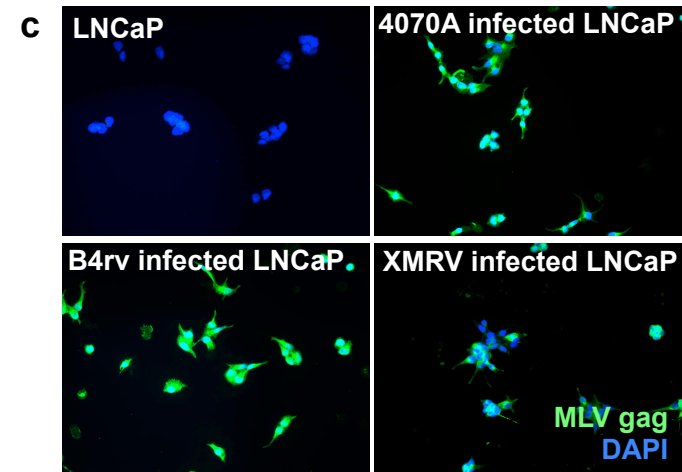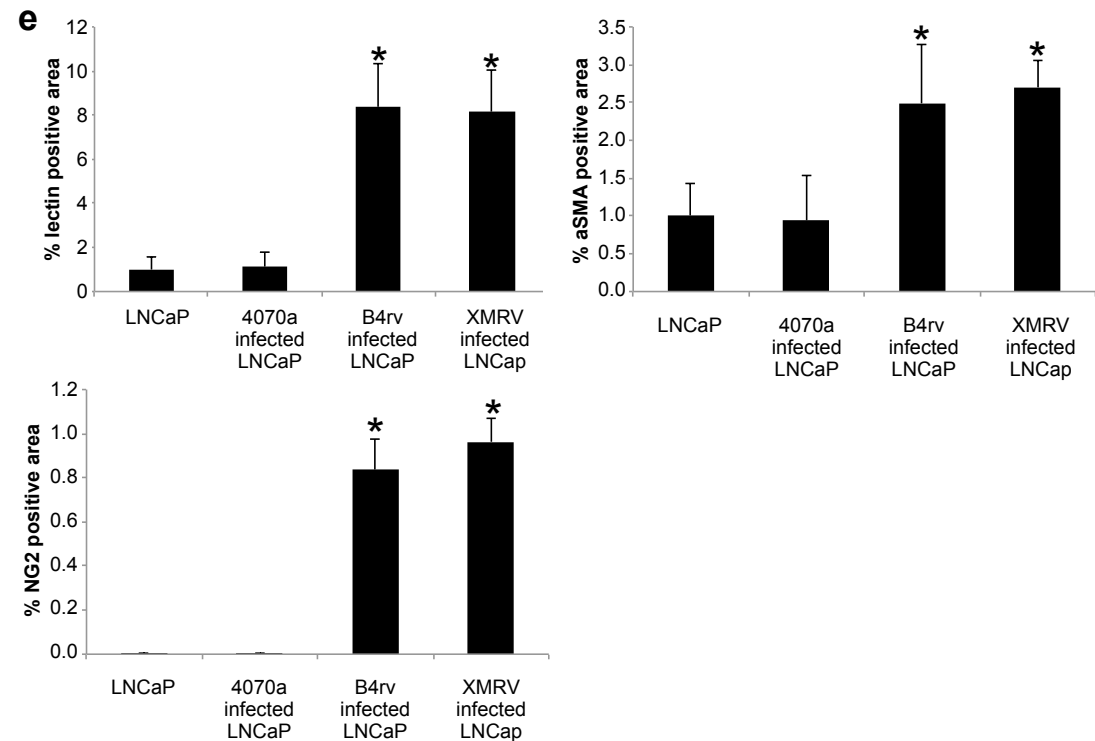

Supplement: Additional file 1: Figure S1 — Schematic describing the generation of xenograft derived cell lines (Thalmann et al.[16]), validation of xenotropic MLV infection and vascular effects in vivo. (A) Adapted from Thalmann et al.[16] describing the generation of the C2-4 B4 cell line from which B4rv was discovered.LNCaP cells and a human bone fibroblast (MS) cell line, were injected into a nude mouse to generate a primary subcutaneous tumor, after which the mouse was castrated. The subsequent androgen independent tumor was established as the C4 cell line. An additional mouse was castrated and injected with C4 and MS cells to generate the C4-2 cell line. The C4-2 cell line was injected into additional mice to generate bone metastasis cell lines including C4-2 B2, C4-2 B3/B5 and the C4-2 B4. (B) The B4rv genome represented in red followed by open reading frames (ORFs) as black arrows.ORFs were determined and drawn using Gene Construction Kit(2), with a minimum ORF length of 250 base pairs, ‘ATG’ starting codon and searching only the top strand (5’ to 3’). The B4rv genome was isolated from genomic DNA of infected C4-2 B4 cell line via PCR using primers described in Urisman et. Al.(3) (see Table 1) and sequenced on an ABI 3730 DNA Analyzer, 750-1000 base pairs at a time. Overlapping sequences were assembled using the Geneious software(4) package. (C) In vitro staining with an antibody to MLV-gag, in 4070a-, B4rv- or XMRV-infected LNCaP cells infected with 1x10^6^ infectious units, and no gag staining of the control LNCaP cells. (D) Reverse transcriptase activity in 4070a-, B4rv- or XMRV-infected, or uninfected LNCaP cells. (E) Quantification of tumor sections (n=10) stained with an endothelial cell specific lectin, alpha-SMA antibody, NG2 antibody. Measurements were taken by determining the percent of each field that was positive for signal using ImageJ(5). (F) A representative image of nude mice with tumors resulting from injection of LNCaP cells infected with 4070a (left) or XMRV (right) at 12 weeks post-i [file 1742-4690-10-34-S1.pdf]

**Fig. S2**

**a**

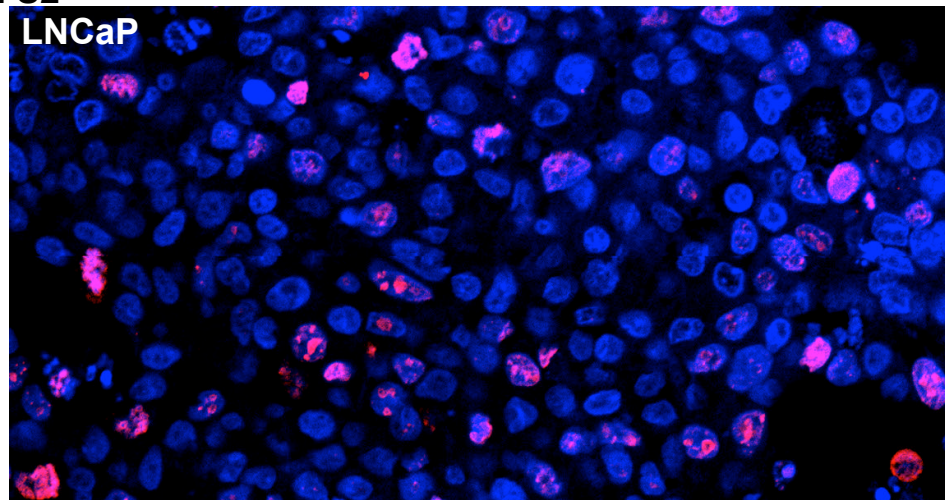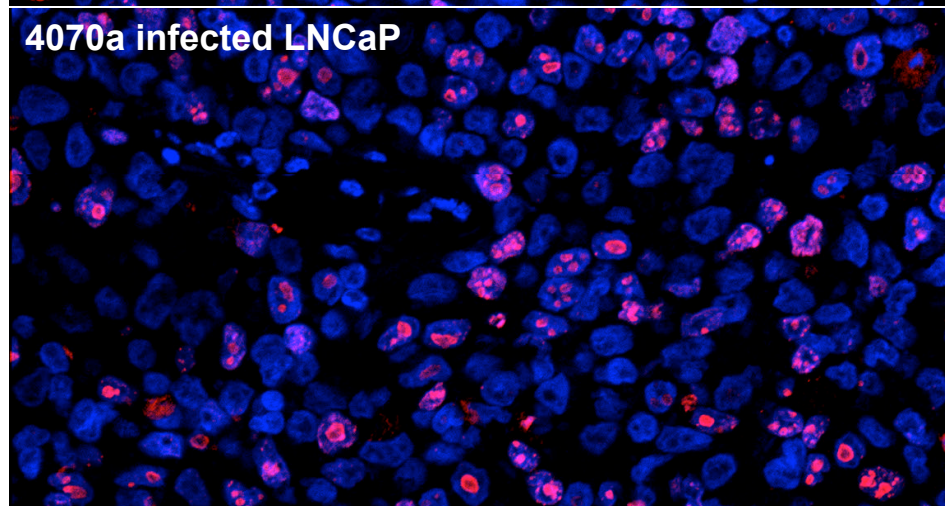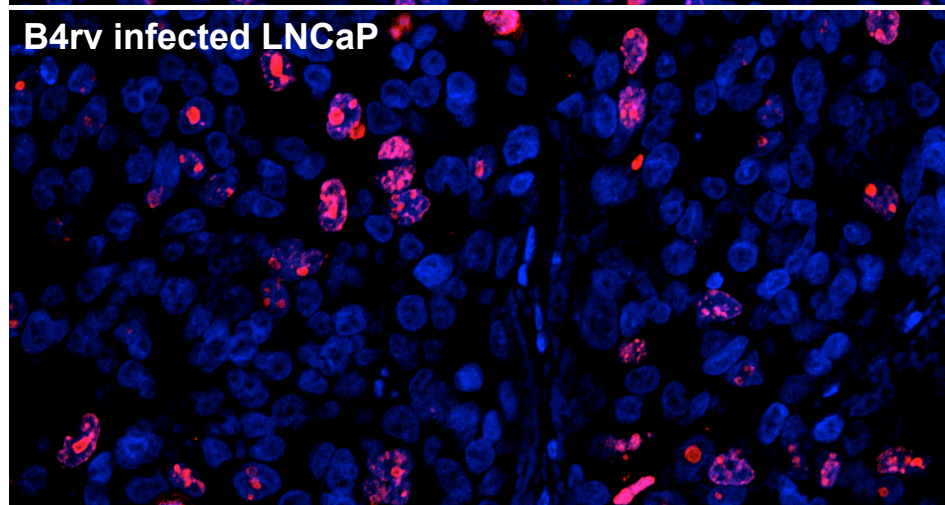

**b**

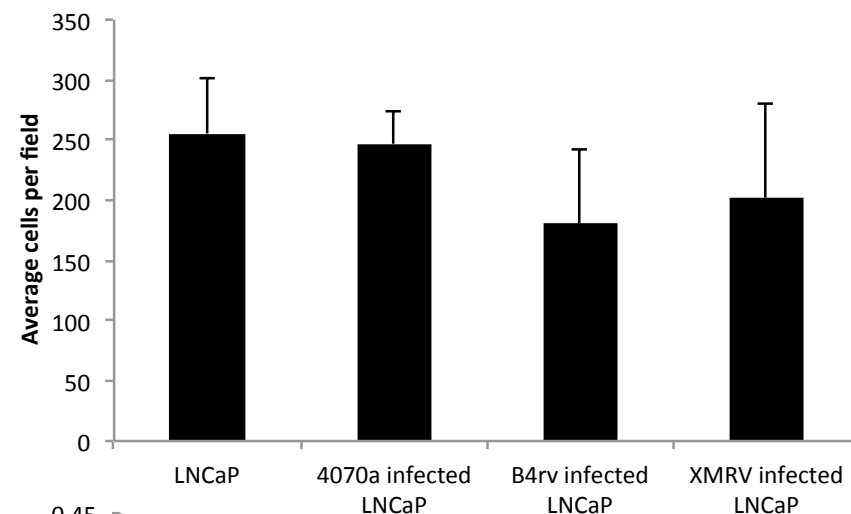

**c**

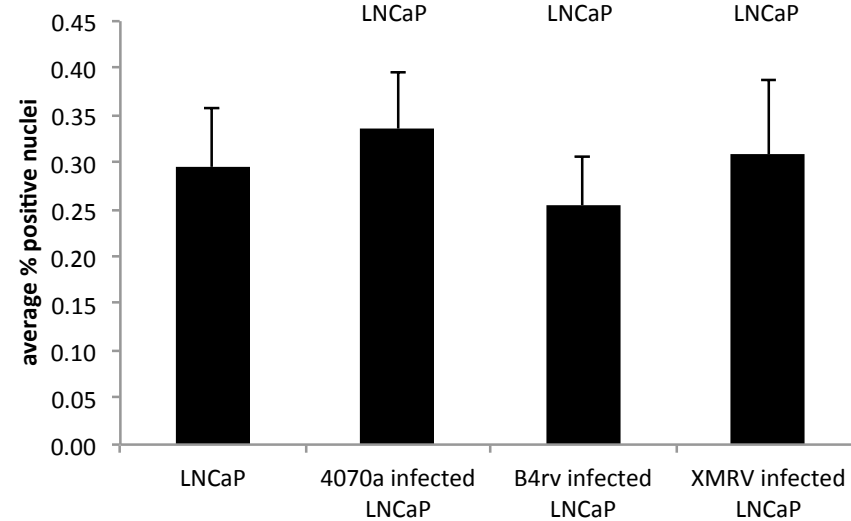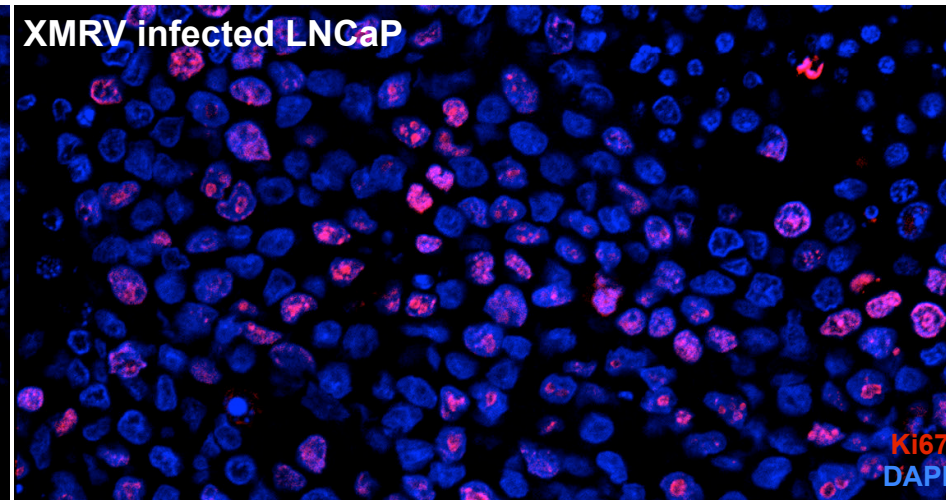

Supplement: Additional file 2: Figure S2 — LNCaP cells infected with MoMLV-4070a, B4rv or XMRV do not exhibit an increase in proliferation above uninfected LNCaP cells. (A) Representative Ki-67 staining of paraffin-embedded tumor sections resulting from subcutaneous injection of LNCaP cells infected with either 4070a, B4rv or XMRV into nude mice. (B) Quantification of in vitro proliferation assay where LNCaP cells infected with 4070a, B4rv or XMRV and the uninfected LNCaP control were plated at 1x10^6^ cells per well, grown for 24 hours, then stained with crystal violet and counted at 20x. Results represent an average of 3 fields observed per well, 2 wells per group. (C) Quantification of in vivo Ki-67 staining displayed in (A), where positive nuclei were counted from sections of 10 tumors per group. [file 1742-4690-10-34-S2.pdf]

**Fig. S3**

**a**

**LNCaP**

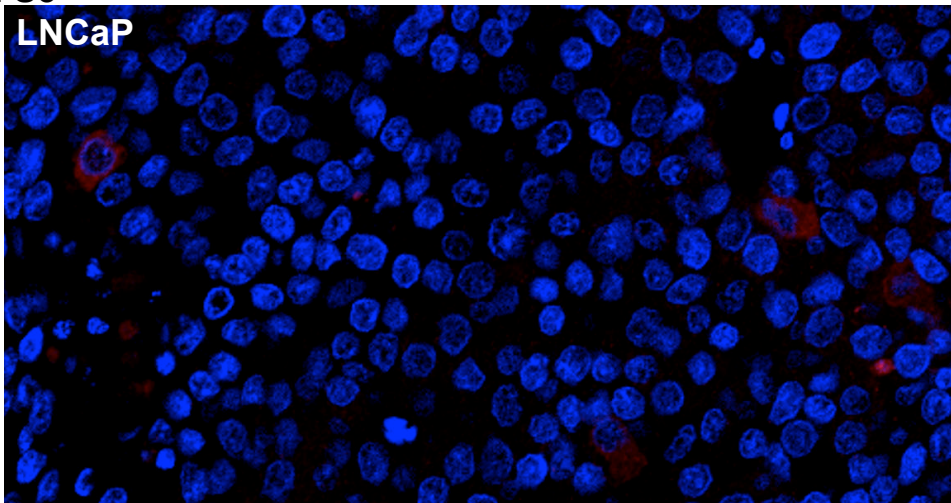

**4070a infected LNCaP**

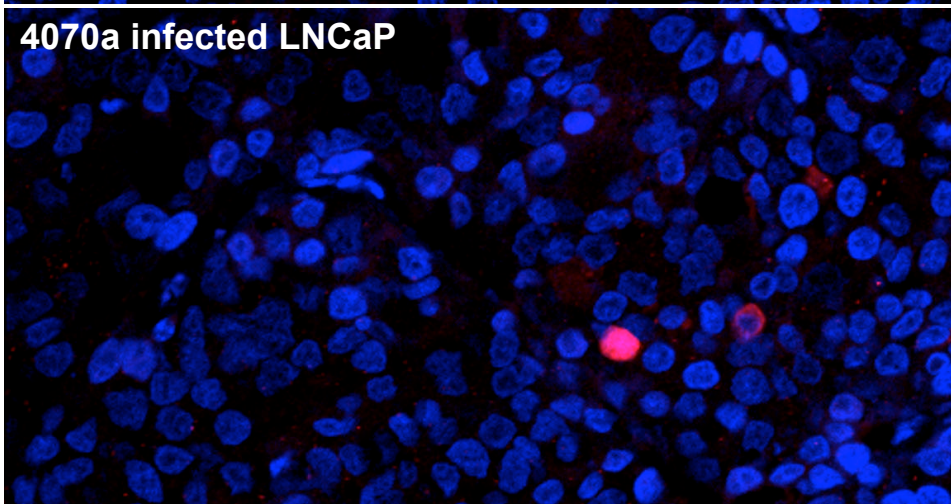

**B4rv infected LNCaP**

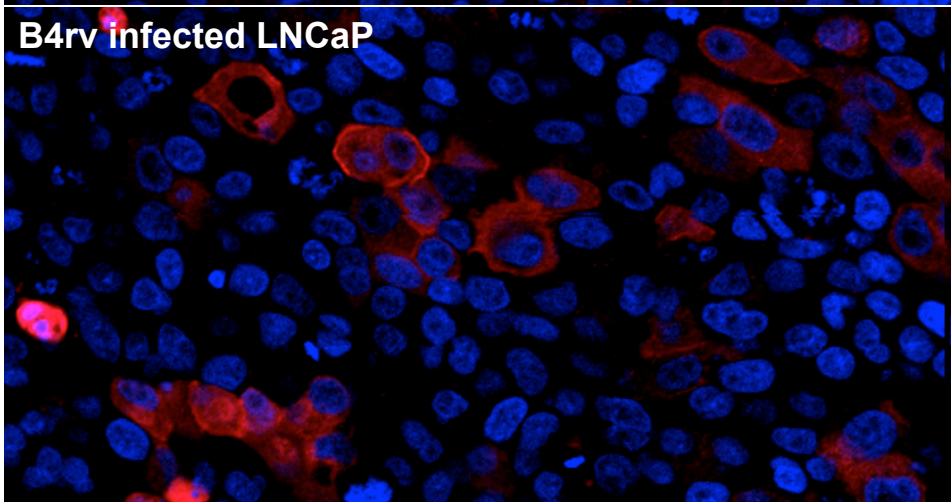

**b**

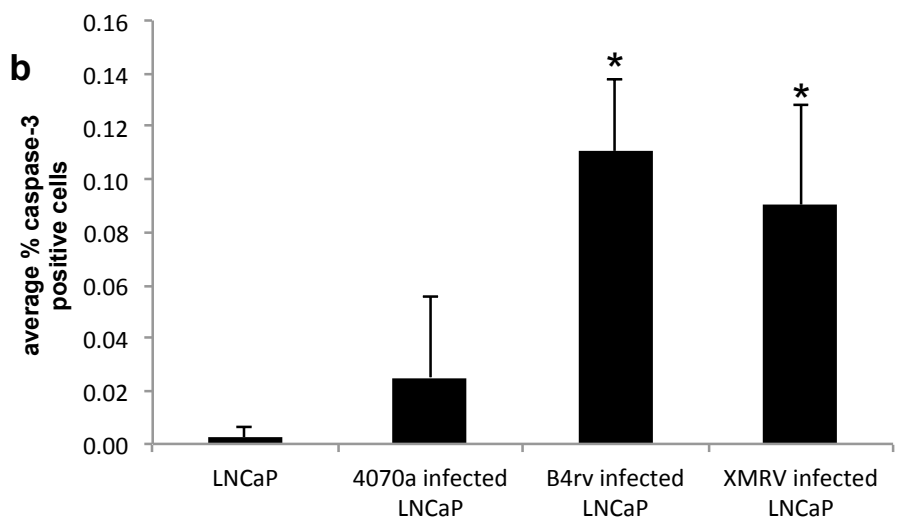

**XMRV infected LNCaP**

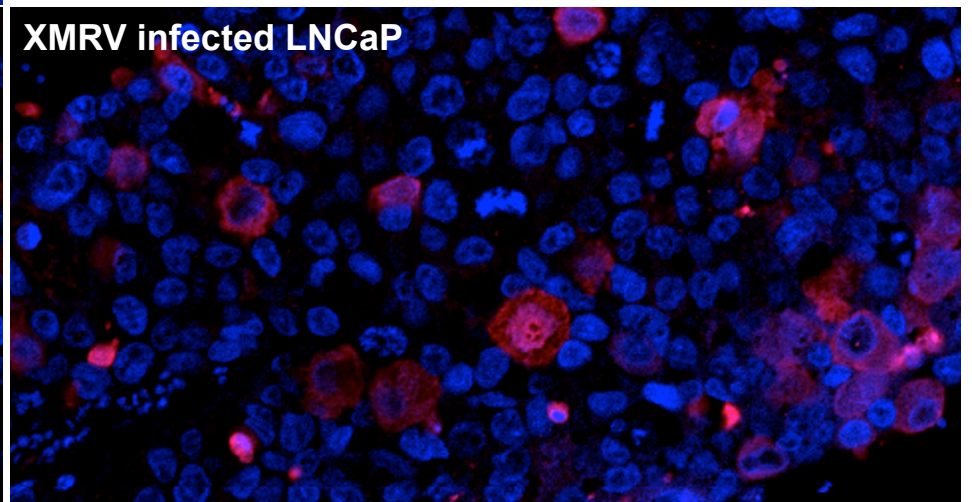

Supplement: Additional file 3: Figure S3 — LNCaP cells infected with MoMLV-4070a, B4rv or XMRV exhibit an increase in apoptosis compared to uninfected LNCaP cells. (A) Representative cleaved caspase-3 staining of paraffin-embedded tumor sections resulting from subcutaneous injection of LNCaP cells infected with either 4070a, B4rv or XMRV into nude mice. (B) Quantification of in vivo cleaved caspase-3 staining displayed in (A), where positive nuclei were counted from sections of 10 tumors per group. [file 1742-4690-10-34-S3.pdf]

Fig. S4

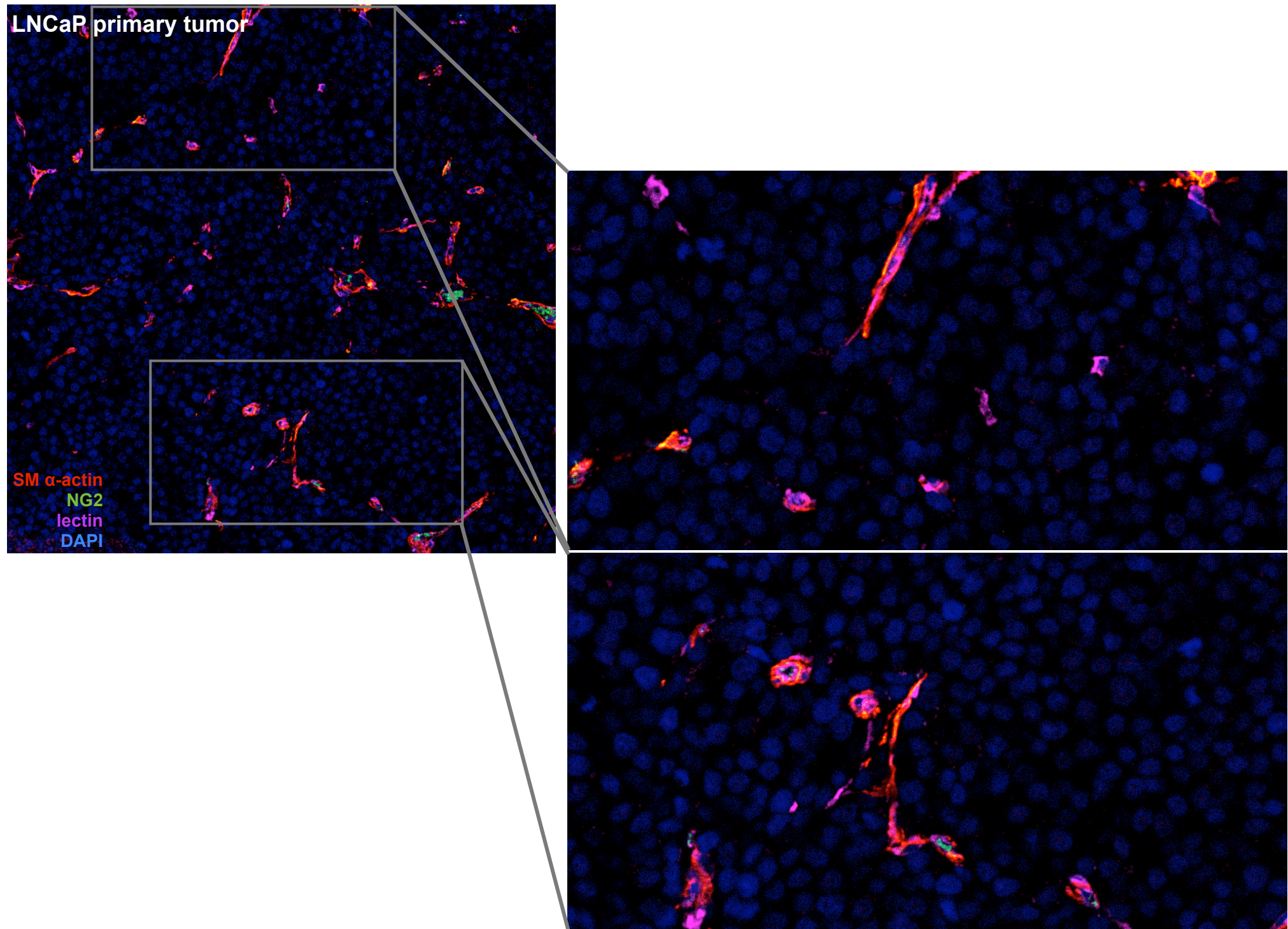

Supplement: Additional file 4: Figure S4 — Representative confocal image of an uninfected LNCaP tumor stained for vascular cell markers. Merged confocal image showing staining of a paraffin-embedded tumor section resulting from subcutaneous injection of uninfected LNCaP cells into a nude mouse, shown at 20x. Insets are shown at 120% digital zoom using the Zeiss Zen 2009 software. SM α-actin, an SMC marker is shown in red, NG2, a pericyte marker, is shown in green, isolectin for endothelial cells is shown in magenta and nuclear stain DAPI is shown in blue. [file 1742-4690-10-34-S4.pdf]

Fig. S5

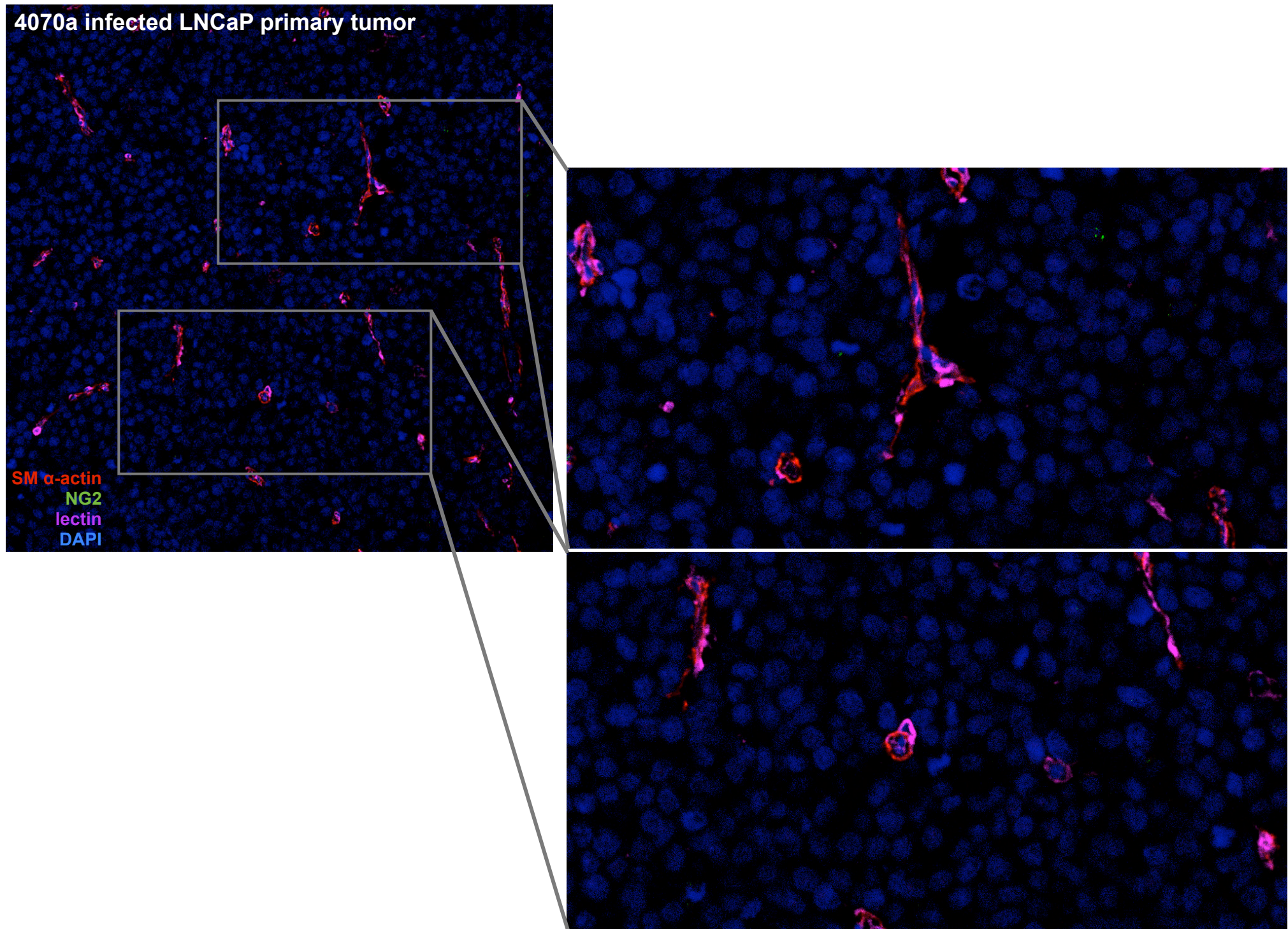

Supplement: Additional file 5: Figure S5 — Representative confocal image of a MoMLV-4070a infected LNCaP tumor stained for vascular cell markers. Merged confocal image showing staining of a paraffin-embedded tumor section resulting from subcutaneous injection of 4070a infected LNCaP cells into a nude mouse, shown at 20x. Insets are shown at 120% digital zoom using the Zeiss Zen 2009 software. SM α-actin, an SMC marker is shown in red, NG2, a pericyte marker, is shown in green, isolectin for endothelial cells is shown in magenta and nuclear stain DAPI is shown in blue. [file 1742-4690-10-34-S5.pdf]

Fig. S6

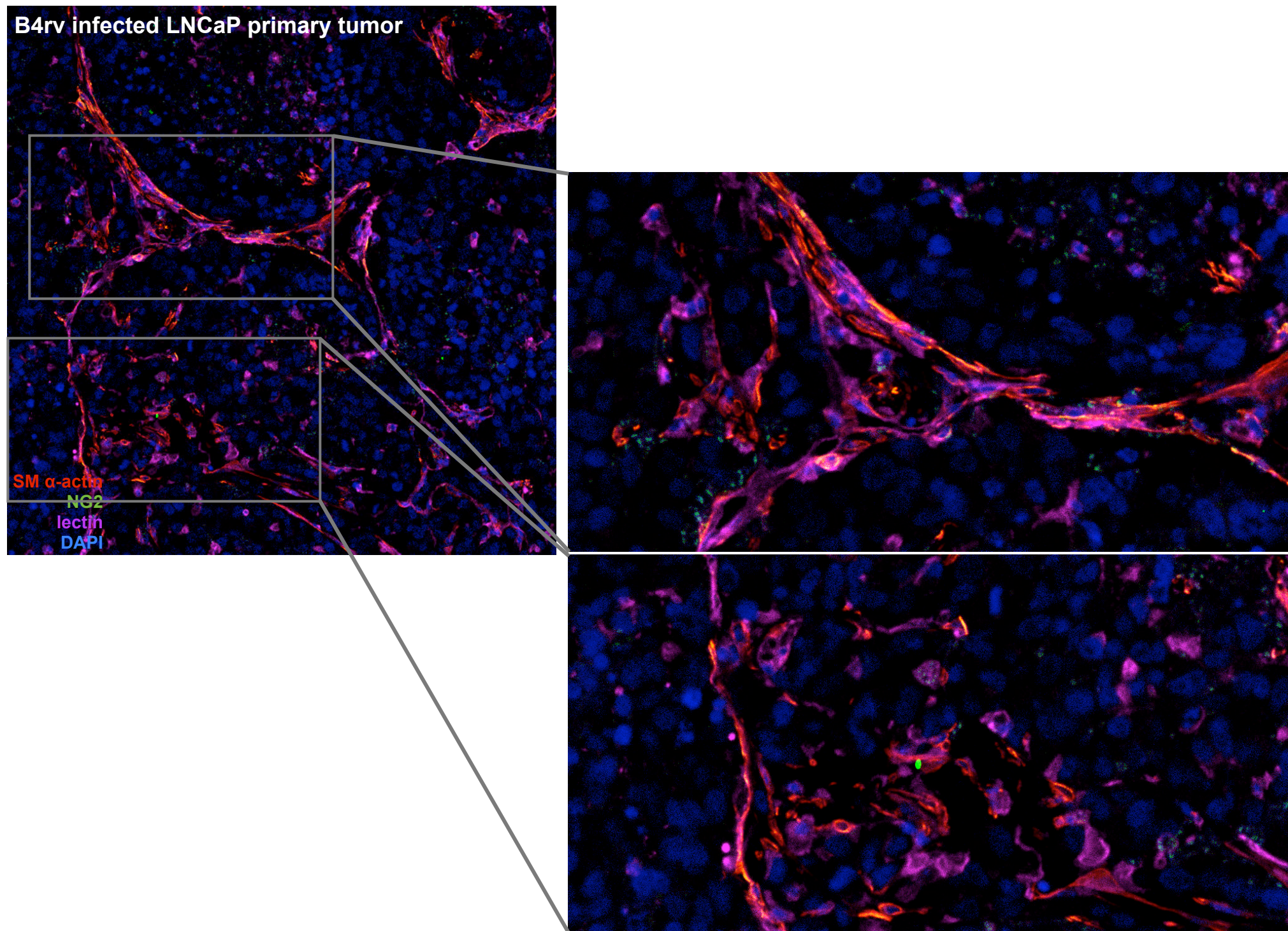

Supplement: Additional file 6: Figure S6 — Representative confocal image of a B4rv infected LNCaP tumor stained for vascular cell markers. Merged confocal image showing staining of a paraffin-embedded tumor section resulting from subcutaneous injection of B4rv infected LNCaP cells into a nude mouse, shown at 20x. Insets are shown at 120% digital zoom using the Zeiss Zen 2009 software. SM α-actin, an SMC marker is shown in red, NG2, a pericyte marker, is shown in green, isolectin for endothelial cells is shown in magenta and nuclear stain DAPI is shown in blue. [file 1742-4690-10-34-S6.pdf]

Fig. S7

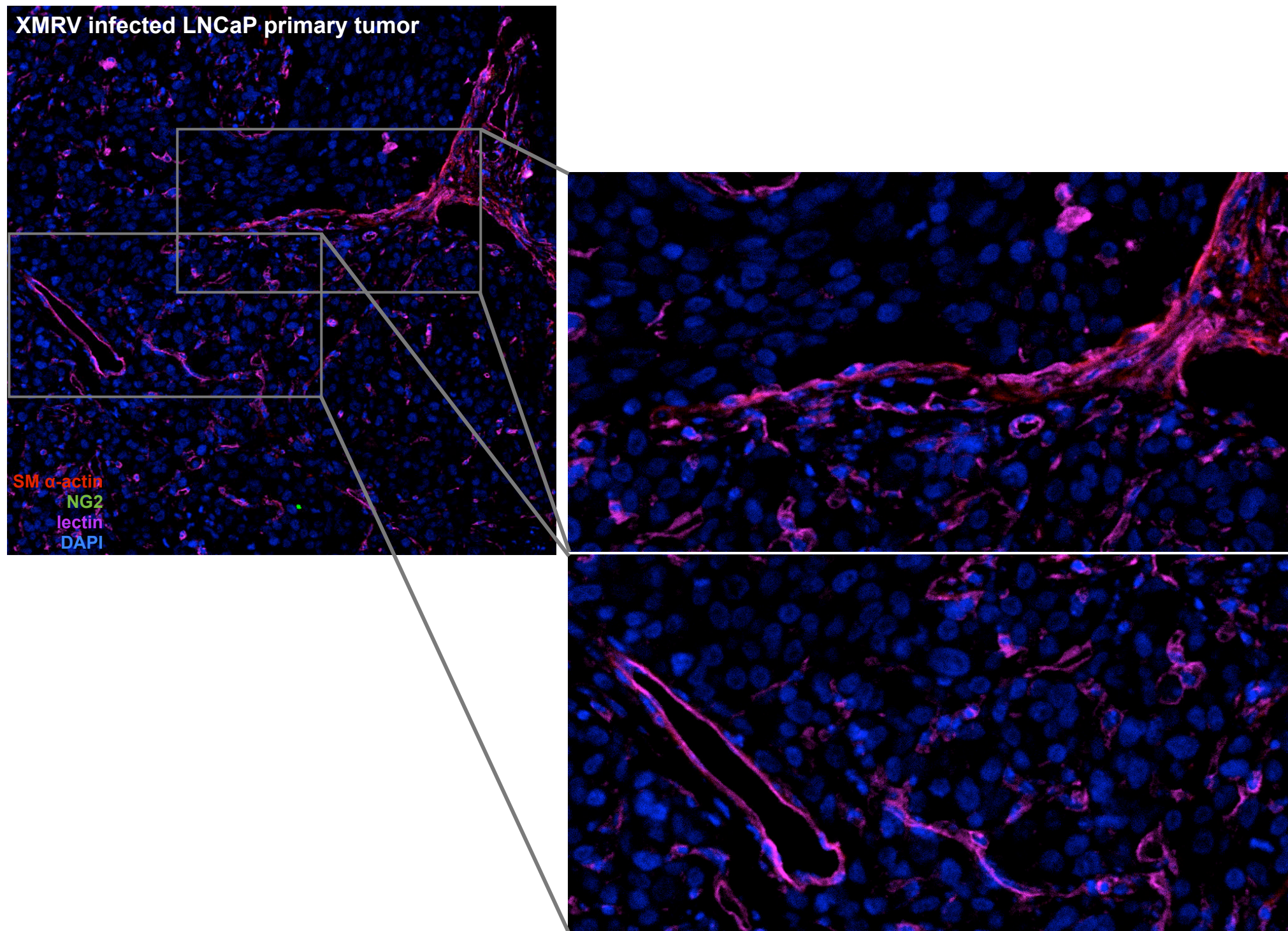

Supplement: Additional file 7: Figure S7 — Representative confocal image of an XMRV infected LNCaP tumor stained for vascular cell markers. Merged confocal image showing staining of a paraffin-embedded tumor section resulting from subcutaneous injection of XMRV infected LNCaP cells into a nude mouse, shown at 20x. Insets are shown at 120% digital zoom using the Zeiss Zen 2009 software. SM α-actin, an SMC marker is shown in red, NG2, a pericyte marker, is shown in green, isolectin for endothelial cells is shown in magenta and nuclear stain DAPI is shown in blue. [file 1742-4690-10-34-S7.pdf]

**Fig. S8**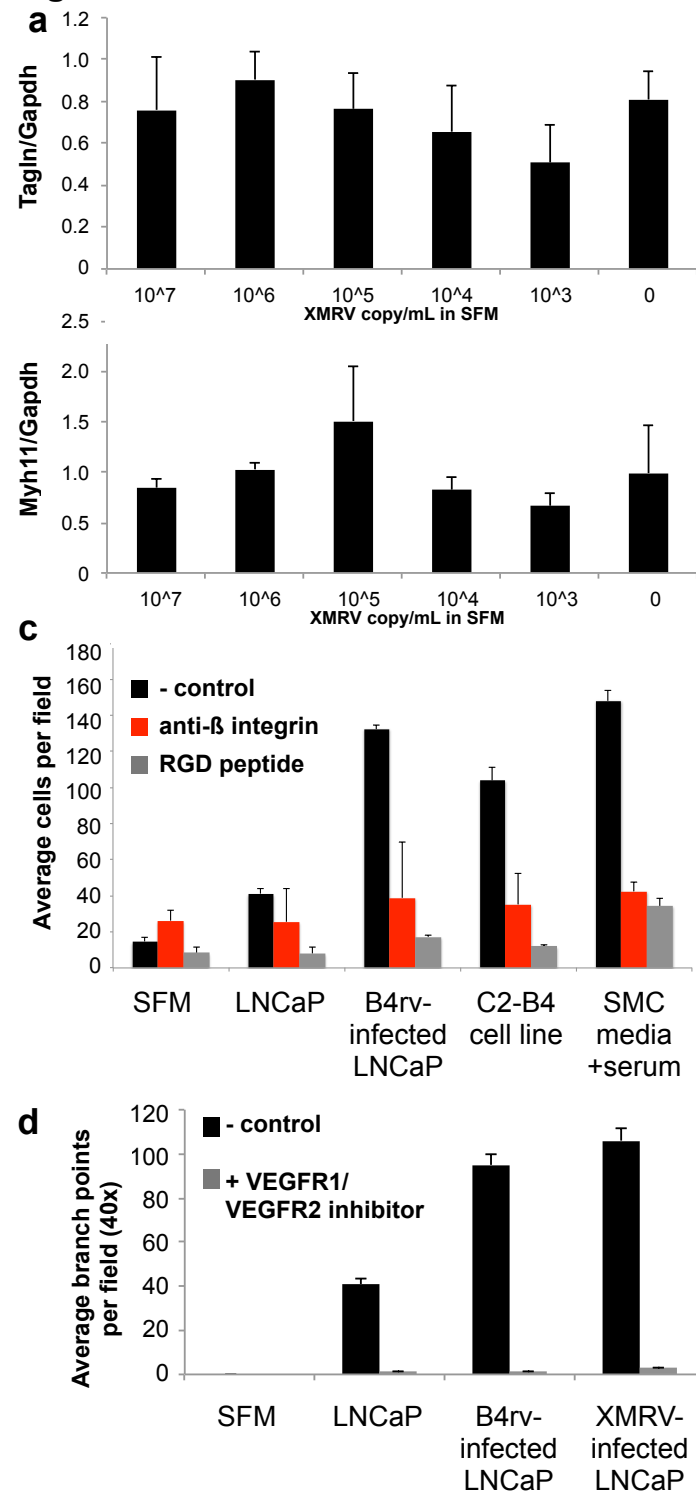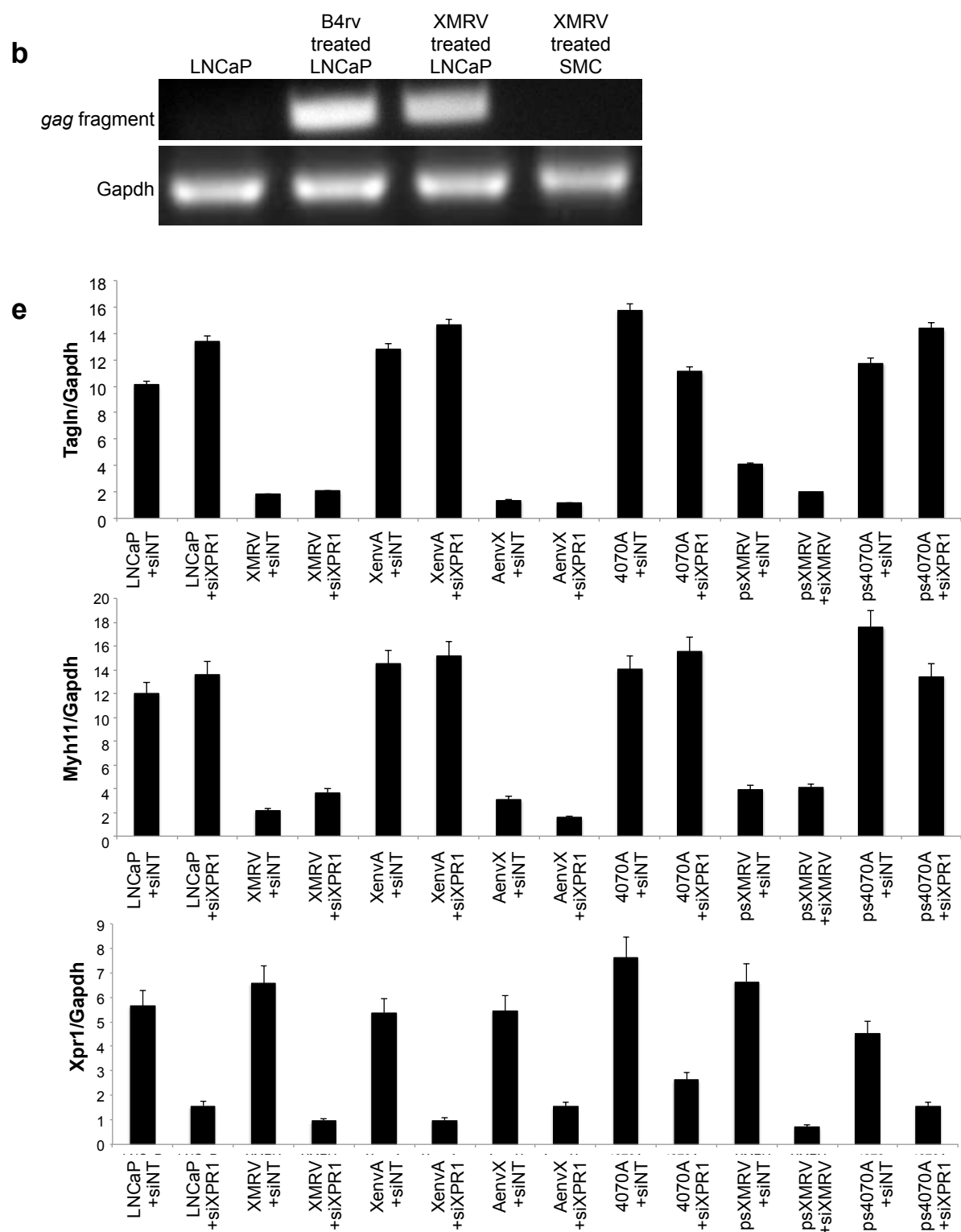

Supplement: Additional file 8: Figure S8 — B4rv or XMRV infection of tumor cells promotes the production of pro-angiogenic soluble factors in vitro, but does not infect vascular SMCs, nor directly suppresses SMC marker gene expressions (A) Q-rtPCR results of rat aortic SMCs Tagln and Myh11 gene expression levels after exposure to XMRV viral particles at a range of concentrations (x-axis). (B) Gel electrophoresis image of PCR for an MLV-gag sequence, using genomic DNA from uninfected LNCaP cells, B4rv treated LNCaP cells, XMRV treated LNCaP cells and XMRV treated SMCs. PCR for GAPDH was used as a loading control. (C) Quantification of SMC migration assay where conditioned media was incubated with a blocking antibody to ß1 integrins or RGD peptides prior to plating under transwell membranes. Membranes were stained with crystal violet 16 hours post-incubation and counted at 40x. (D) Effects of TCM on tube formation by human umbilical cord endothelial cells (HUVEC) with and without addition of a VEGFR1/VEGFR2 inhibitor to the TCM prior to incorporation in matrigel plugs. Endothelial tube branch points from 8–10 randomly chosen high-power fields (magnification x40) were counted. (E) Q-rtPCR results of rat aortic SMCs Tagln, Myh11 and Xpr1 gene expression levels after exposure to conditioned media of LNCaP cells treated first with siRNA to XPR1 or a non-targeted control, then XMRV, 4070a, chimeric viruses 4070a-Xenv or XMRV-Aenv, or pseudovirions expressing GFP and coated with the envelope proteins of either XMRV or 4070a (psXMRV, ps 4070a). [file 1742-4690-10-34-S8.pdf]

**Fig. S9**

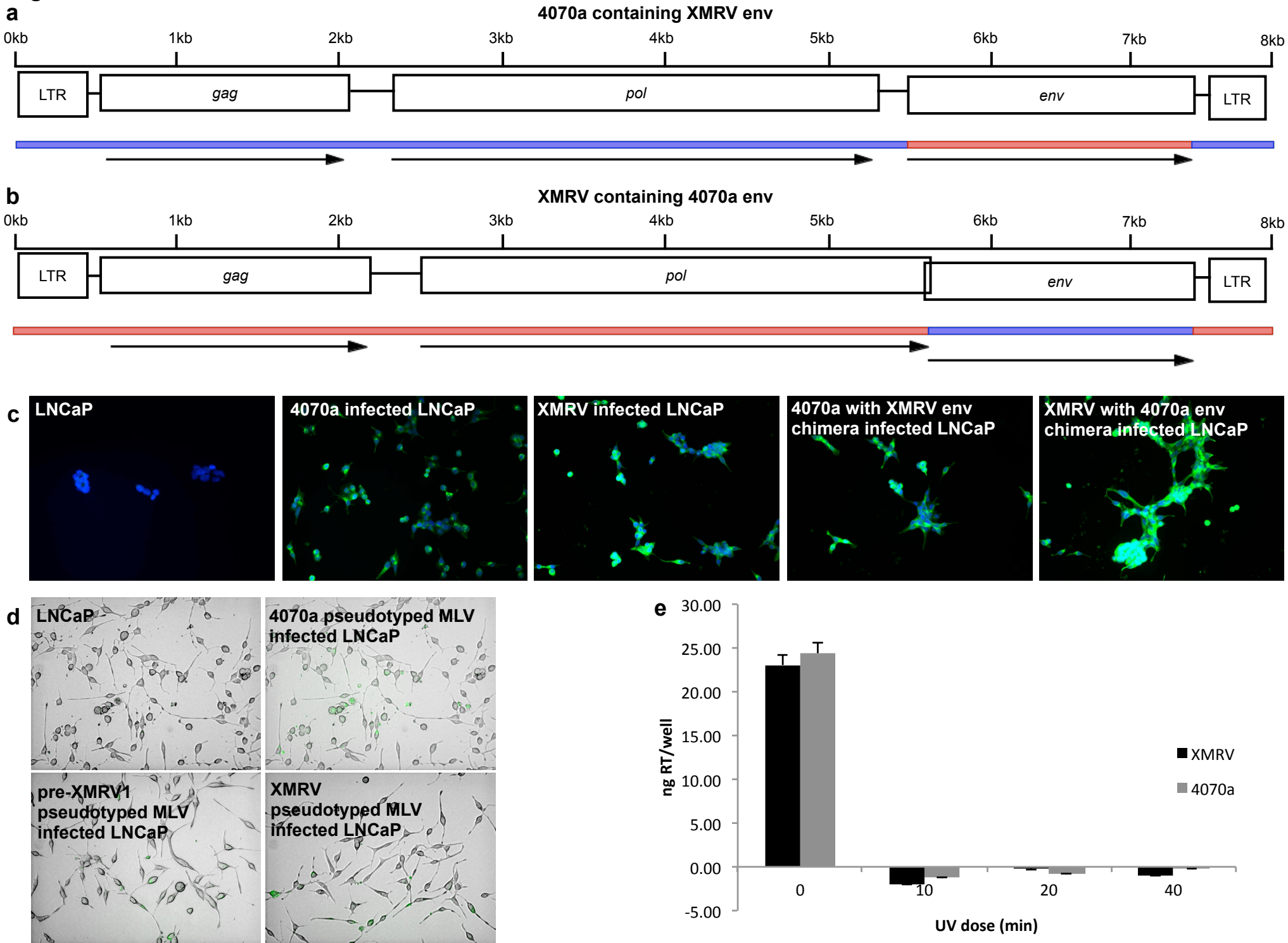

Supplement: Additional file 9: Figure S9 — Rat aortic SMCs are not infected by XMRV in vitro, and direct application of viral particles onto SMCs in culture does not result in the suppression of smooth muscle marker gene expression. (A) Schematic depicting 4070a-Xenv, blue representing the sequence from MoMLV-4070a, and red representing the sequence from XMRV VP62 and black arrows representingORFs. ORFs were determined and drawn using Gene Construction Kit(2), with a minimum ORF length of 250bp, ‘ATG’ start codon and searching only the top strand (5’ to 3’). The 4070a-Xenv chimera was constructed by replacing the env gene of MoMLV-4070a with the env gene of XMRV VP62 via overlap extension (see Table 1) and sequenced at the University of Virginia DNA Sciences Core on an ABI 3730 DNA Analyzer. Overlapping sequences were assembled using the Geneious software(4) package. (B) Schematic depicting XMRV-Aenv,blue representing the sequence derived from MoMLV-4070a, red representing the sequence derived from XMRV VP62 and black arrows representingORFs. ORFs were determined and drawn using Gene Construction Kit(2), with a minimum ORF length of 250bps=, ‘ATG’ start codon and searching only the top strand (5’ to 3’). The XMRV-Aenv chimera was constructed by replacing the env gene of XMRV VP62 with the env gene of MoMLV-4070a via overlap extension (see Table 1) and sequenced at the University of Virginia DNA Sciences Core on an ABI 3730 DNA Analyzer, 750-1000 base pairs at a time. Overlapping sequence contigs were assembled using the Geneious software package(4). (C) In vitro staining for MLV-gag, demonstrating gag protein production in 4070a-, XMRV-, 4070a-Xenv-, or XMRV-Aenv-infected LNCaPs, and no gag staining of control LNCaPs. (D) GFP signal overlayed on bright-field images of LNCaP cells in vitroexposed to MLV viral particles pseudotyped with the envelope of 4070a, pre-XMRV1 or XMRV for 24 hours. (E) Reverse transcriptase detection demonstrating absence of activity by 4070a or XMRV viral particles that were UV irrad [file 1742-4690-10-34-S9.pdf]
